# Application of Adaptive Neuro-Fuzzy Inference System-Non-dominated Sorting Genetic Algorithm-II (ANFIS-NSGAII) for Modeling and Optimizing Somatic Embryogenesis of Chrysanthemum

**DOI:** 10.3389/fpls.2019.00869

**Published:** 2019-07-05

**Authors:** Mohsen Hesami, Roohangiz Naderi, Masoud Tohidfar, Mohsen Yoosefzadeh-Najafabadi

**Affiliations:** ^1^Department of Horticultural Science, Faculty of Agriculture, University of Tehran, Karaj, Iran; ^2^Department of Plant Biotechnology, Faculty of Science and Biotechnology, Shahid Beheshti University, Tehran, Iran; ^3^Department of Plant Agriculture, University of Guelph, Guelph, ON, Canada

**Keywords:** artificial intelligence, chrysanthemum, carbohydrate, embryogenesis, *in vitro* culture, light quality, optimization algorithm, plant growth regulator

## Abstract

A hybrid artificial intelligence model and optimization algorithm could be a powerful approach for modeling and optimizing plant tissue culture procedures. The aim of this study was introducing an Adaptive Neuro-Fuzzy Inference System- Non-dominated Sorting Genetic Algorithm-II (ANFIS-NSGAII) as a powerful computational methodology for somatic embryogenesis of chrysanthemum, as a case study. ANFIS was used for modeling three outputs including callogenesis frequency (CF), embryogenesis frequency (EF), and the number of somatic embryo (NSE) based on different variables including 2,4-dichlorophenoxyacetic acid (2,4-D), 6-benzylaminopurine (BAP), sucrose, glucose, fructose, and light quality. Subsequently, models were linked to NSGAII for optimizing the process, and the importance of each input was evaluated by sensitivity analysis. Results showed that all of the R^2^ of training and testing sets were over 92%, indicating the efficiency and accuracy of ANFIS on the modeling of the embryogenesis. Also, according to ANFIS-NSGAII, optimal EF (99.1%), and NSE (13.1) can be obtained from a medium containing 1.53 mg/L 2,4-D, 1.67 mg/L BAP, 13.74 g/L sucrose, 57.20 g/L glucose, and 0.39 g/L fructose under red light. The results of the sensitivity analysis showed that embryogenesis was more sensitive to 2,4-D, and less sensitive to fructose. Generally, the hybrid ANFIS-NSGAII can be recognized as a powerful computational tool for modeling and optimizing in plant tissue culture.

## Introduction

Chrysanthemum is one of the most important ornamental plants that have high commercial potential due to its diverse floral colors that can be used as cut flowers or pot plants (da Silva and Kulus, 2014). Due to increasing demand for cut flowers, there is high attention for introducing rare and unique varieties through genetic engineering (Noda et al., 2017). Therefore, it is necessary to establish a rapid and cost-effective micro-propagation system for shortening the breeding cycle and genetic manipulation of this valuable plant. Also, one of the prerequisites for gene transformation systems is the high ability whole plants regeneration from *in vitro* culture. Through somatic embryogenesis, a large number of transgenic plants and elite clones can be propagated over a short period (Naing et al., 2013).

Although the successfulness of the somatic embryogenesis on chrysanthemum has been published in several publications (May and Trigiano, 1991; Pavingerová et al., 1994; Tanaka et al., 2000; da Silva, 2003; Shinoyama et al., 2004; Mandal and Datta, 2005; Xu et al., 2012; Naing et al., 2013), there is a lack of effort on increasing the capacity of somatic embryogenesis and the number of somatic embryos per explant. Several factors can be influenced on the efficiency of somatic embryogenesis, such as the type of explants, basal medium, growth regulators, carbohydrate source, and physical environment (light and temperature) of the culture (da Silva, 2003). Murashige and Skoog (MS) medium (Murashige and Skoog, 1962) has been reported as an appropriate medium for embryogenesis of chrysanthemum (May and Trigiano, 1991; Tanaka et al., 2000; da Silva, 2003; Xu et al., 2012; Naing et al., 2013). MS medium is a plant growth medium used in the laboratories for plant cell, tissue, and organ culture. MS was invented by plant scientists Murashige and Skoog (1962). The type and concentration of plant growth regulators (PGRs) for using in the culture medium are important to achieve efficient somatic embryogenesis (da Silva, 2003). Also, the combination of 2,4-dichlorophenoxyacetic acid (2,4-D) and 6-benzylaminopurine (BAP) is critical in somatic embryogenesis of chrysanthemum (da Silva, 2003). May and Trigiano (1991) examined the effect of light and darkness, as well as different sucrose concentrations on embryogenesis of leaf segments of chrysanthemum and obtained 4.1% embryogenesis in MS medium containing 1.0 mg/L 2,4-D and 0.2 mg/L BAP along with 12% sucrose in light condition. In another study, Pavingerová et al. (1994) tested different concentrations of PGRs on embryogenesis of chrysanthemum but they did not mention the capacity of somatic embryogenesis. Tanaka et al. (2000) studied the effect of various concentrations of PGRs on embryogenesis of ray florets of chrysanthemum and reported 58% of somatic embryogenesis that was obtained from MS medium supplemented with 57.08 μM Indole-3-acetic acid (IAA) and 0.46 μM kinetin. Mandal and Datta (2005) also reported the effects of different concentrations of 2,4-D and BAP on direct somatic embryogenesis from ray florets of different chrysanthemum cultivars and reported 40% embryogenic response on MS medium consisting of 4 mg/L 2,4-D and 2 mg/L BAP. Shinoyama et al. (2004) tested different PGRs concentrations on somatic embryogenesis of leaf segments in chrysanthemum and reported that the highest embryogenesis (21.3%) was achieved in MS medium supplemented with 2 mg/L 2,4-D along with 1 mg/L kinetin. Xu et al. (2012) described the effects of different concentrations of BAP and 1-naphthaleneacetic acid (NAA) on indirect somatic embryogenesis of chrysanthemum via leaf and stem segments. They reported that the best somatic embryogenesis response (93%) was obtained from leaf explant in MS medium along with 1.5 mg/L NAA and 0.5 mg/L BAP. Naing et al. (2013) examined the effect of different concentrations of PGRs and sucrose on direct embryogenesis of leaf segments of chrysanthemum and obtained 100% embryogenesis in MS medium containing 2.0 mg/L 2,4-D and 2.0 mg/L BAP along with 3% sucrose in light condition.

It is a general stand that specific carbohydrates might have various effects on morphogenesis during plant tissue culture. Though different mechanisms and aspects of carbon metabolism are well-described, there is a lack of comprehensive studies on the relationship of carbohydrates to growth responses in plant tissue culture (Yancheva and Roichev, 2005). The three most important carbohydrates are sucrose, glucose, and fructose that broadly used for plant cells grown under *in vitro* culture (Fuentes et al., 2000).

Another factor that is of high paramount to robustify the cells plant tissue growth and the biosynthesis of metabolites is the varying bands of the light spectrum (Kendal et al., 2013). Previous studies have shown the effectiveness of using light emitting diodes (LEDs) as the lighting source during plant tissue culture (Li et al., 2010; Kendal et al., 2013; Nhut et al., 2015; Chen et al., 2016; Ferreira et al., 2017). Such these LEDs can be used for adjusting the photosynthetically active radiation (PAR) and photomorphogenic radiation rates (PRR) that would be vital for plant growth and development (Nhut et al., 2015). Therefore, providing such studies on the effectiveness of specific bands of LEDs on different species would be helpful for increasing the efficiency of plant tissue culture (Ferreira et al., 2017). The plant responses against various bands of LEDs would be described as anatomical, morphological, and physiological responses such as changes in leaf anatomy, shoots and somatic embryo formation, and the improvement in the photosynthetic abilities of plants cultivated under *in vitro culture* conditions (Li et al., 2010; Kendal et al., 2013; Nhut et al., 2015; Ferreira et al., 2017). However, there is a lack of study for the interaction of different factors involved in somatic embryogenesis so it is necessary to schedule an investigational plan for comprehensive studying in the embryogenesis.

Plant biological systems consist of non-linear and non-deterministic developmental processes (Gago et al., 2010b). Genetics, the environment and their interaction are three of the most fundamental factors cause to complexity of plant growth and development systems. These three key factors contain a high level of inconsistency among and in them that they ultimately cause a unique developmental process. A similar circumstance can be found in plant tissue culture (Gago et al., 2010b; Prakash et al., 2010). The conventional analytical techniques for the solving problems of the plant tissue culture are not appropriate for modeling non-linearity in the complex systems (Gago et al., 2011; Arab et al., 2016, 2018; Jamshidi et al., 2016; Nezami-Alanagh et al., 2017; Niazian et al., 2018). Nowadays, the area of data-driven modeling, especially artificial intelligence models such as neural networks and fuzzy logic, have presented an appropriate alternative to model the non-linearity and ill-defined systems found in the plant tissue culture (Hesami et al., 2017; Arab et al., 2018; Nezami-Alanagh et al., 2018). In a modeling study, the artificial neural network (ANN) approach was applied for modeling the effects of nitrate concentrations, sucrose contents, fresh weight, size, and some explants per vessel, pH, temperature, the volume of growth medium, and time of inoculation in hairy root culture (Prakash et al., 2010). ANN was used for the modeling of medium compositions for shoot proliferation of *Prunus* rootstocks (Arab et al., 2016), *in vitro* rooting *Prunus* rootstocks (Arab et al., 2018), shoot proliferation of pear rootstocks (Jamshidi et al., 2016), embryogenesis of ajowan (Niazian et al., 2018). Gago et al. (2010a) utilized neuro-fuzzy logic approach for modeling the effects of different types of auxin and sucrose concentrations on *in vitro* root formation and acclimatization of *Vitis vinifera* L. In another modeling study (Gago et al., 2014), the effects of light and sucrose concentrations on *in vitro* rooting of *V. vinifera* were modeled by neuro-fuzzy logic approach.

The fuzzy systems, broadly applied for understanding system behavior, are very interpretable (Łapa et al., 2018). The fuzzy systems are able to model human knowledge by way of understandable linguistic terms. Since this system is not plausible when there is no specialist available, a primary knowledge base can be ready using fuzzy IF–THEN rules by a specialist (Li et al., 2018). On the other hand, neural networks possess good learning capabilities but they lack the interpretation capability (Łapa et al., 2018). Therefore, the neuro-fuzzy system as a powerful method that has both interpretability and learning capabilities, is the result of the combination of fuzzy logic and neural network (Gago et al., 2014). Recently, various neuro-fuzzy approaches have been designed and developed. Among them, an adaptive neuro-fuzzy inference system (ANFIS) gives a directed and systematic approach to modeling and provides the best possible design parameters in the minimum time (Łapa et al., 2018).

The performance of the plant tissue culture systems in optimization problems can be evaluated by objective functions. There are many trials and errors to optimize the inputs. Recent studies have used a genetic algorithm (GA) in plant tissue culture to reduce computational volumes (Arab et al., 2016, 2018; Jamshidi et al., 2016). GA, as one of the well-known evolutionary computational based optimization algorithms, causes to achieve optimal solutions by using an intelligent approach relying on bio-inspired operators such as selection, crossover, and mutation (Jamshidi et al., 2016; Arab et al., 2018). On the other hand, plant tissue culture problems have to satisfy various objective functions by considering different constraints. However, GA as a single-objective algorithm cannot optimize multi-objective functions, simultaneously (Bozorg-Haddad et al., 2016; Hosseini-Moghari et al., 2017). Therefore, the multi-objective algorithm has been required for the optimization process (Hesami et al., 2019b). Classical optimization methods consist of multi-criterion decision-making methods providing the converting model of the multi-objective optimization problem to a single-objective optimization problem by emphasizing one particular Pareto-optimal solution at a time. Considering this method for multiple solutions, it has to be applied so many times for finding various solutions at each simulation run (Bozorg-Haddad et al., 2016). The Non-dominated Sorting Genetic Algorithm-II (NSGA-II) has been known as the first evolutionary multi-objective optimization algorithms, try to find the solution domain for discovering Pareto-optimal solutions within a multi-objective centered scheme (Wang et al., 2018).

In this study, we tried to model the somatic embryogenesis by using ANFIS. We linked the model to NSGAII to find the maximum efficiency and the optimum conditions, which are essential for significant *in vitro* embryogenesis. Therefore, this study was aimed to model and optimize the appropriate hormonal combinations, carbohydrate sources, and light quality for somatic embryogenesis of chrysanthemum via ANFIS-NSGAII.

## Materials and Methods

### Plant Materials

The leaf explants of chrysanthemum “Hornbill Dark” were collected from grown greenhouse mother plants. The leaf explants were washed with tap water for 30 min and washed again after cleaning with a liquid soap solution. Additional surface sterilization was applied in a laminar air flow chamber. The explants were sterilized with 70% aqueous ethanol for 40 s, dipped 15 min in 1.5% (v/v) NaOCl solution, and washed three times with sterilized distilled water. Afterward, 25 mm^2^ leaf segments (abaxial side) were incubated on 200-ml glass flasks containing 40 ml basal medium.

### Media and Culture Condition

MS medium as a basal medium was used in this experiment having 0.7% agar (Duchefa Biochemie, Netherlands). Following the applying of treatments (different types and concentrations of carbohydrate sources and PGRs), the pH of the medium was adjusted to 5.8 using 1 N KOH or 1 N HCl before autoclaving at 121°C for 20 min. All cultures were kept at 25 ± 2°C under 16-h photoperiod with the light intensity of 50 μmol m^−2^s^−1^.

### Experimental Design

The experiments were conducted in a completely randomized design (CRD) with the factorial arrangement, and there were 15 replicates per treatment, and each treatment was repeated in three sets.

In order to develop the protocol of somatic embryogenesis for chrysanthemum, different factors were studied as follows:

1. Effects of combination of 2,4-D (0.5, 1.5, and 2.5 mg/L) and BAP (0.5, 1.5, and 2.5 mg/L) on callogenesis frequency (CF), embryogenesis frequency (EF), and the number of somatic embryo (NSE).
2. Effects of sucrose (2, 3, and 6%), glucose (3, 6, and 9%), and fructose (3, 6, and 9%) alone or in combination (2% sucrose + 2% glucose + 2% fructose;) on CF, EF, and NSE.
3. Effects of different LED light qualities (red, blue and white) and darkness on CF, EF, and NSE. Due to the fact that the inputs in the modeling should be quantitative, the colors of the LED lamps were quantitated by using the RGB (Red, Green, Blue) coding. LED treatments were as follows:

- Darkness: without light (RGB = 0,0,0),
- White: 100% white light (RGB = 255,255,255),
- Blue: 100% blue light with a wavelength of 460 nanometers (RGB = 0,0,255),
- Red: 100% red light with a wavelength of 660 nanometers (RGB = 255,0,0).

After 6 weeks of culturing, the efficiency of growth factors on embryogenesis was determined by recording CF, EF, and NSE.

### ANFIS Model

To construct ANFIS model, 2,4-D, BAP, sucrose, glucose, fructose, R, G, and B were considered as inputs, and CF, EF, and NSE were considered as outputs data for the modeling of growth factors. Based on a trial and error approach, the Gaussian membership function was considered (between 3 and 5 membership function for different variable). Also, the number of epochs for training the models set to 10. Moreover, 75 and 25% of the dataset were used to train and test the models, respectively. The dataset was checked for confirming the range of the train set containing the test data. To determine and improve the performance of the best-constructed model, various values for significant model's parameters were tested based on a trial and error analysis. Finally, for each model, the best-resulted output with the minimum estimation error was determined based on the coefficient of determination (R^2^), Root Mean Square Error (RMSE), and Mean Bias Error (MBE) as follows:

(1) $$\begin{array}{l} R^{2}=\;1-\frac{\sum_{i=1}^{n}{\left(y_{i}-{ŷ}_{i}\right)}^{2}}{\sum_{i=1}^{n}{\left(y_{i}-{ȳ}_{i}\right)}^{2}}\left(0\leq R^{2}\leq 1\right) \end{array}$$

(2) $$\begin{array}{l} RMSE=\sqrt{\left(\sum_{i=1}^{n}{\left(y_{i}-{ŷ}_{i}\right)}^{2}\right)/n}\left(0\leq RMSE\leq +\infty \right) \end{array}$$

(3) $$\begin{array}{l} MBE=1/n\sum_{i=1}^{n}\left(y_{i}-{ŷ}_{i}\right)\left(-1\leq MBE\leq +1\right) \end{array}$$

Where *n* is the number of data, *y*_*i*_is the value of predicted datasets, and ŷ_*i*_ is the value of average observed datasets. Best fit can be indicated in the case that RMSE values closer to 0 and R^2^ values closer to 1. The MBE value stands for negative and positive calculation error, indicating the similarity of the predicted values with observational values.

The overall equation of ANFIS model can be determined as follow:

(4) $$\begin{array}{l} \begin{array}{c} \mathrm{Rule}\;1:\mathrm{if}\;x\;\mathrm{is}\;A_{1}\;\mathrm{and}\;y\;\mathrm{is}\;B_{1}\;\mathrm{then}\;z_{1}=p_{1}x+q_{1}y+r_{1}\\ \mathrm{Rule}\;2:\mathrm{if}\;x\;\mathrm{is}\;A_{1}\;\mathrm{and}\;y\;\mathrm{is}\;B_{2}\;\mathrm{then}\;z_{2}=p_{2}x+q_{2}y+r_{2}\\ \mathrm{Rule}\;3:\mathrm{if}\;x\;\mathrm{is}\;A_{2}\;\mathrm{and}\;y\;\mathrm{is}\;B_{1}\;\mathrm{then}\;z_{3}=p_{3}x+q_{3}y+r_{3}\\ \mathrm{Rule}\;4:\mathrm{if}\;x\;\mathrm{is}\;A_{2}\;\mathrm{and}\;y\;\mathrm{is}\;B_{2}\;\mathrm{then}\;z_{4}=p_{4}x+q_{4}y+r_{4} \end{array} \end{array}$$

**Figure 1:**
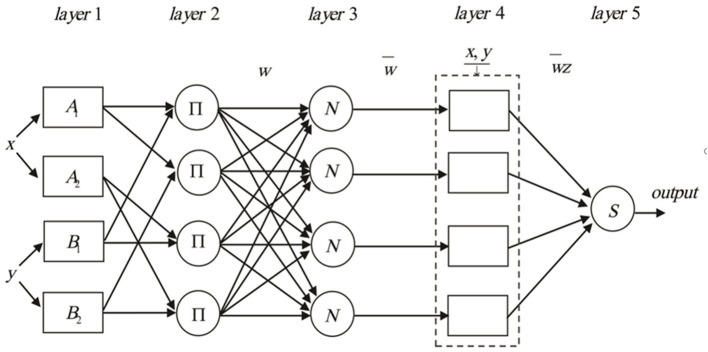
The schematic diagram of the proposed ANFIS methodology.

Where *x* and *y* are inputs, and *z* is output, *A*_*i*_ and *B*_*i*_ (i = 1,2,3,4) are the fuzzy sets, *p*_*i*_,*q*_*i*_ and *r*_*i*_(i = 1,2,3,4) are the design parameters that are determined during the training process. The construction of ANFIS can broadly be classified as five layers consisting of input nodes, rule nodes, average nodes, following nodes, and output nodes layers (Figure 1). The input, output, and node functions of each layer are as follows:

*layer 1*: *input nodes (adaptive nodes) layer*. The layer one outputs represent the fuzzy membership grade of the inputs, which are defined by:

(5) $$\begin{array}{l} \begin{array}{c} O_{i}^{1}=\mu_{A_{i}}\left(x\right)\;i=1,\;2.\\ O_{i}^{1}=\mu_{B_{i-2}}\left(x\right)\;i=3,\;4. \end{array} \end{array}$$

Where $O_{i}^{1}$: output from node *i* and μ : membership functions.

*layer2*: *rule nodes layer*. Each node in the second layer is considered as a fixed node labeled ∏. The AND operator is implemented to achieve an output that gives the result of the before that rule. The output of the *k*^th^ node (*w*_*k*_) is defined as:

(6) $$\begin{array}{l} O_{k}^{2}=w_{k}=\mu_{A_{i}}\left(x\right)\mu_{B_{j}}\left(x\right)\;i=1,\;2.\;j=1,\;2.\;k=1,\;2,\;3\;4. \end{array}$$

Which represents the rule's firing strength.

*layer3*: *average nodes layer*. Each node in this layer is a fixed node labeled *N*. The function of this layer is the calculation of the ratio of the *i*th rule's firing strength in the second layer. The outputs of this layer ($\bar{w_{i}}$) are called normalized firing strengths, can be computed as:

(7) $$\begin{array}{l} O_{i}^{3}=\bar{w_{i}}=\frac{w_{i}}{\overset{4}{\underset{l=1}{\sum }}w_{k}}\;i=1,\;2,\;3\;4. \end{array}$$

*layer4*: *consequent nodes layer*. In this layer, the contribution of each *i*th rules into the total output is computed. The output of each node in this layer is simply the product of the normalized firing strength and a first order Sugeno model. Thus, the outputs of the fourth layer can be defined as:

(8) $$\begin{array}{l} O_{i}^{4}=\bar{w_{i}}z_{i}=\bar{w_{i}}\left(p_{i}x+q_{i}y+r_{i}\right)\;i=1,\;2,\;3\;4. \end{array}$$

*layer5*: *output nodes layer*. In this layer, there is only one single fixed node labeled *S*. The task of the fifth layer is a summation of all incoming signals. Hence, the final output of the ANFIS model is given by:

(9) $$\begin{array}{l} O_{i}^{5}=\overset{4}{\underset{i=1}{\sum }}\bar{w_{i}}z_{i} \end{array}$$

It can be observed that there are two sets of parameters that should be adjusted. The first set is related to the input membership functions, which are the so-called premise parameters. The second set is three parameters related to first order Sugeno model. These parameters are the so-called consequent parameters. The least-squares method is used to optimize the consequent parameters, and backpropagation algorithm is applied to adjust the premise parameters not separately but rather in combination. It has been proven that a hybrid algorithm has high efficiency in training the ANFIS (Farokhnia et al., 2011).

### Optimization Process (NSGA-II)

In order to select the best non-dominated solutions via a step-by-step procedure, NSGA-II should mainly depend on binary tournament selection, elitist non-dominated sorting, and crowding distance. The computational process should be started by the initialization of the chromosome/population. Mutation operations, selection, and cross-over are three main components for simulation process that can be useful for evaluating objective functions and decision variables.

Afterward, the solutions, which are not dominated by the others, categorized as different non-dominated fronts of the population are derived based on the non-dominated sorting concept. Each non-dominated front can be sorted as a rank or level data, and the population is ranked again except for the first Pareto front. Therefore, the non-dominated front considered as the first rank is the last generation of the optimal Pareto. The latest procedure is to remove members that possess the highest rank (lower priority) and then select others to generate parent population of the next generation.

Afterward, each objective function should be estimated by the crowding distance of a specific solution. Crowding distance is based on the average of two related neighboring solutions. Considering the lowest density of solutions that have less priority, the solutions of each level are categorized by crowding distance in descending order.

**Figure 2:**
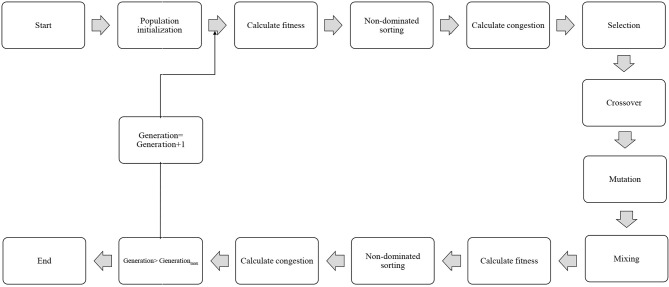
Schematic diagram showing the step-by-step NSGAII optimization process.

The next step after sorting the solutions is a selection step. The binary tournament selection operator is commonly used in the selection step. Therefore, a solution with greater crowding distance and lower rank will be chosen between two random solutions derived from the population/chromosome. Thus, the child population is generated based on repeating the selection operator with applying the mutation operators and cross-over, the same as the exact size of the parent population. Finally, the non-dominated sorting is utilized for the combination of child and parent populations after performing a simulation process for estimating the objective functions. The optimal solutions of each generation produce a new parent population during the last step “elitism” that final derived solution is known as the optimal Pareto front (Figure 2). In this study, EF and NSE were considered as two objective functions to determine the optimum values of inputs. The ideal point of Pareto was chosen such that EF and NSE became the maximum. In other words, a point in the Pareto front was considered as the solution such that:

(10) $$\sqrt{{\left(EF-m\right)}^{2}+{\left(NSE-n\right)}^{2}}$$

was minimal; where *m* and *n* are the maximum EF (%) and NSE in observed data, respectively.

### Sensitivity Analyses

The sensitivity CF, EF, and NSE against the investigating growth elements was evaluated by using the following criterion;

The variable sensitivity error (VSE) value stands for the overall performance of the developed ANFIS model in the case that the particular independent variable is not available.

Variable sensitivity ratio (VSR) value: If all variables are available, VSR demonstrates the correlation between the error of the ANFIS model and VSE.

The higher the VSR, the more important variable will be. Therefore, all input variables can be ranked based on their importance.

The mathematical code was written conveniently for Matlab (version 9.5) software to construct and assess the models.

### Validation Experiment

During the validation experiment, the growth factors optimized by ANFIS-NSGAII were tested to evaluate the efficiency of ANFIS-NSGAII for modeling and optimizing the growth factors for embryogenesis parameters (i.e., EF and NSE).

## Results

While many studies have focused on the effect of PGRs and sucrose concentrations in somatic embryogenesis of chrysanthemum, there is a lack of such comprehensive studies on the influence of carbohydrate sources, PGRs, and light quality. The light and carbohydrate sources, as well as PGRs, are essential factors in plant tissue culture that dramatically influence the somatic embryogenesis. In this study, the interaction effects of 2,4-D, BAP, sucrose, fructose, glucose, and light quality on callogenesis frequency (CF), embryogenesis frequency (EF), and number of somatic embryo (NSE) of chrysanthemum were investigated.

The results indicated that CF and EF were achieved in MS medium containing both 2,4-D and BAP. However, no callus formation, as well as somatic embryogenesis, was observed on MS medium with low concentration (0.5 mg/l) of PGRs. Within 2 and 3 weeks of inoculation, callus induction and embryogenesis were observed at the cut ends of the leaf segments, respectively. By increasing the concentration of PGRs (up to 1.5 mg/L), EF and NSE were increased significantly. Also, the highest CF, EF, and NSE were observed in the combination of 1.5 mg/L 2,4-D and 1.5 mg/L BAP (Table S1).

Different results in CF, EF, and NSE were achieved among the cultures under different light qualities. The results of the effects of light quality on embryogenesis parameters showed that the best efficiency was obtained under red light (Table S1). Also, acceptable somatic embryogenesis was achieved in darkness compared with white or blue light. However, the lowest EF and NSE was observed in blue light treatment (Table S1).

Furthermore, type and concentration of carbohydrate had a significant effect on CF, EF, and NSE. Thus, the highest CF, EF, and NSE were observed in the 6% glucose. Glucose as monosaccharides had a better impact than sucrose as a disaccharide on embryogenesis parameters. Although monosaccharides at a higher level (up to 6%) were appropriate for embryogenesis, sucrose at lower concentration (3%) was more useful for this purpose (Table S1).

Generally, the highest NSE was achieved in MS medium supplemented with 1.5 mg/L 2,4-D and 1.5 mg/L BAP along with 6% glucose under red light (Table S1).

### ANFIS Modeling and Evaluation

ANFIS was used for modeling the three outputs (CF, EF, and NSE) based on eight variables including 2,4-D, BAP, sucrose, glucose, fructose, R, G, and B.

**Figure 3:**
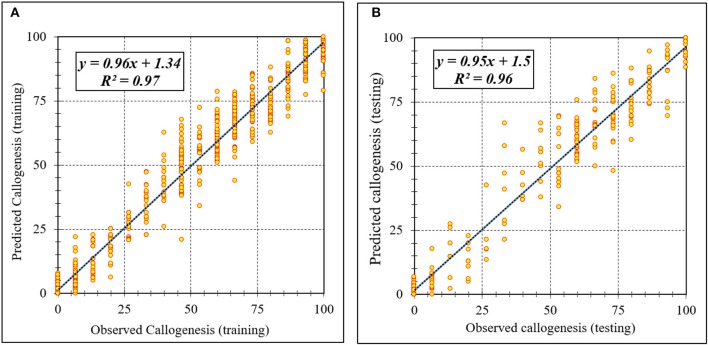
Scatter plot of model predicted vs. observed values of callogenesis of chrysanthemum obtained by ANFIS model. **(A)** Training set (*n* = 810); **(B)** Testing set (*n* = 270). Fitted simple regression line on scatter points was indicated by a solid line.

**Figure 4:**
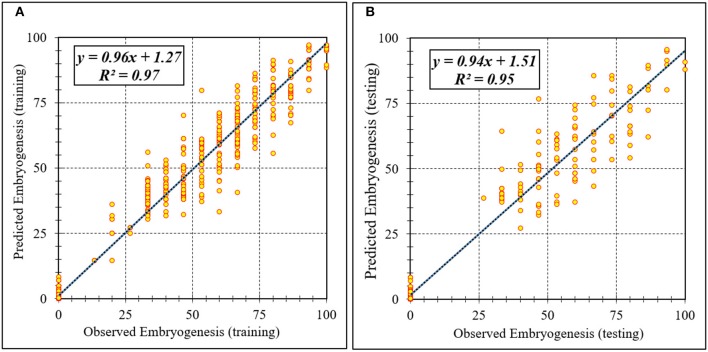
Scatter plot of model predicted vs. observed values of embryogenesis of chrysanthemum obtained by ANFIS model. **(A)** Training set (*n* = 810); **(B)** Testing set (*n* = 270). Fitted simple regression line on scatter points was indicated by a solid line.

**Figure 5:**
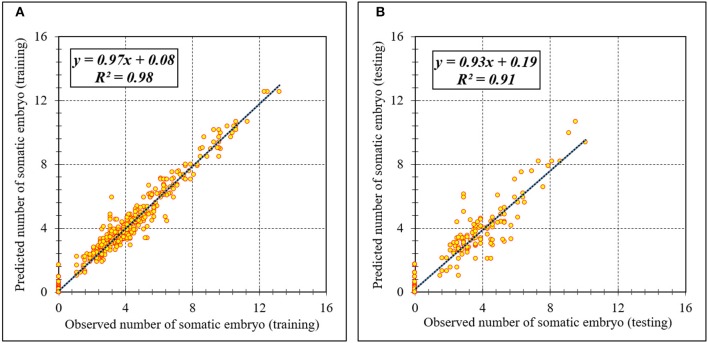
Scatter plot of model predicted vs. observed values of number of somatic embryo of chrysanthemum obtained by ANFIS model. **(A)** Training set (*n* = 810); **(B)** Testing set (*n* = 270). Fitted simple regression line on scatter points was indicated by a solid line.

Assessment of predicted and observed data describes the efficiency of the ANFIS model. According to Table 1, all of the R^2^ of training and testing data was over 91%. As can be seen in Table 1, the ANFIS model was successful in predicting CF, EF, and NSE. Correlations between observed and predicted data for CF, EF, and NSE demonstrated the good fit of the ANFIS model. The graphs (Figures 3–5) may apply to comprehend the perfect growth factors response and to measure the combined effects of growth factors. The ANFIS model could precisely predict CF (R^2^ > 0.96), EF (R^2^ > 0.95), and NSE (R^2^ > 0.91) in the testing processes that were not used throughout the training data sets (Table 1). Furthermore, the trained ANFIS models of CF, EF, and NSE had balanced performance criteria for both phases of training and testing. Generally, performance criteria (Table 1) illustrated that the ANFIS models were able to efficiently fit published data on the performances of somatic embryogenesis to different types and concentrations of PGRs and carbohydrates as well as light quality.

**Table 1:** Statistics of ANFIS models for callogenesis frequency (CF), embryogenesis frequency (EF), and number of somatic embryo (NSE) embryo of chrysanthemum (training vs. testing values).

**Item**	**CF**		**EF**		**NSE**	
	**Training**	**Testing**	**Training**	**Testing**	**Training**	**Testing**
R^2^	0.975	0.956	0.974	0.947	0.980	0.912
RMSE	5.696	7.484	5.354	7.294	0.373	0.686
MBE	−0.032	−0.536	0.324	−0.117	0.029	0.057

### Sensitivity Analysis of the Models

The comparative rank of input data was calculated through the entire 1080 data lines (training and testing) to determine the general VSR. The VSR achieved for the model output (CF, EF, and NSE), with respect to growth factors (Table 2). Analysis of callogenesis showed that CF was more sensitive to 2,4-D, followed by BAP, glucose, sucrose, fructose, R, G, and B (Table 2). In both EF and NSE models, the feed efficiency indicated more sensitivity for 2,4-D, followed by BAP, glucose, sucrose, R, B, G, and fructose (Table 2).

**Table 2:** Importance of growth factors for callogenesis frequency (CF), embryogenesis frequency (EF), and number of somatic embryo (NSE) of chrysanthemum according to sensitivity analysis on the developed ANFIS model to rank the importance of growth factors.

**Output**	**Item**	**2,4-D**	**BAP**	**GLU**	**FRU**	**SUC**	**R**	**G**	**B**
CF	VSR	5.95	3.13	1.43	1.24	1.31	1.17	1.16	1.10
	Rank	1	2	3	5	4	6	7	8
EF	VSR	4.94	3.63	1.49	1.17	1.33	1.30	1.22	1.25
	Rank	1	2	3	8	4	5	7	6
NSE	VSR	6.08	5.80	1.61	0.91	1.52	1.48	1.04	1.41
	Rank	1	2	3	8	4	5	7	6

### Model Optimization

The ultimate purpose of this study was to analyze ANFIS model to provide an accurate answer of what levels of growth factors may be applied to obtain the maximum EF as well as NSE. Thus, we have linked the model to NSGAII for finding the maximum efficiency and the optimum growth factors levels which are essential for significant *in vitro* embryogenesis.

**Figure 6:**
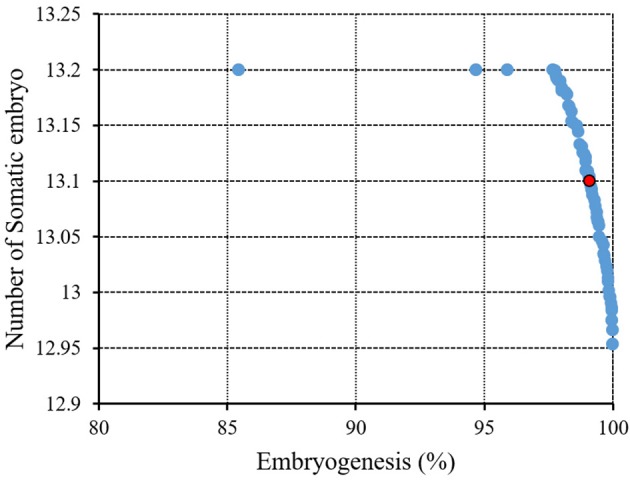
Pareto front obtained by NSGAII for the maximum embryogenesis frequency and number of somatic embryo of chrysanthemum. The red point indicates the ideal point.

Figure 6 and Table 3 showed the results of the optimization process. The lower bound and upper bound of input variables (Table S1) were considered as constraints during the optimization process, and the point with the highest CF and NSE was considered as the ideal point (Figure 3). As can be seen in Table 3, optimal EF (99.1%) and NSE (13.1) can be obtained from a medium containing 1.53 mg/L 2,4-D, 1.67 mg/L BAP, 57.2 g/L glucose, 13.74 g/L sucrose, 0.39 g/L fructose under red light (R.G.B = 254.48, 0.57, 18.25).

**Table 3:** Optimizing growth factors according to optimization analysis on the developed ANFIS-NSGAII in the ideal point for embryogenesis frequency (EF) and number of somatic embryo (NSE) in chrysanthemum.

**Input variable**								**Ideal point of EF**	**Ideal point of NSE**
**2,4-D**	**BAP**	**GLU**	**FRU**	**SUC**	**R**	**G**	**B**		
1.53	1.67	57.20	0.39	13.74	254.48	0.57	18.25	99.10	13.10

### Validation Experiment

The results of the validation experiment (Table 4) showed that ANFIS-NSGAII model could be able to specify the optimal growth factors levels for obtaining the most appropriate results for the studied parameters. The optimized growth factors via ANFIS-NSGAII resulted in 100% EF and 12.83 NSE which a little lower than one predicted.

**Table 4:** Validation of the predicted vs. tested data for embryogenesis frequency (EF) and number of somatic embryo (NSE) of chrysanthemum.

**Treatment**	**EF (%)**	**NSE**
Predicted via ANFIS-NSGAII	99.10	13.10
Tested in validation experiment	100	12.83

## Discussion

Efficient regeneration protocols are necessary for reporting the successful application of genetic transformation approaches (Elhiti et al., 2013). Under this circumstance, the novel and unique genotypes have a chance to be expressed as the plant level (da Silva, 2003). The process of somatic embryogenesis offers a means to propagate a large number of elite clones and transgenic plants over a short period of time (Elhiti et al., 2013). Due to the sensitiveness of the somatic embryos to culture conditions, the conditions of the culture such as the genotype and the explant source, physical environment, and the composition of the medium play important roles during the regeneration and induction of somatic embryos (da Silva, 2003; Elhiti et al., 2013).

In the current study, ANFIS-NSGAII model was used to achieve a useful understanding of the effect of different level of 2,4-D, BAP, sucrose, fructose, and glucose as well as light on somatic embryogenesis of chrysanthemum, and to obtain new insights into improving chrysanthemum embryogenesis condition. According to the best of our knowledge, this study is the first report of using the ANFIS-NSGAII model for modeling and optimizing growth factors for somatic embryogenesis.

A high coefficient of determination between observed and predicted values for both training and testing processes showed the accuracy of the models for the three parameters studied. The high efficiency of neuro fuzzy logic in plant tissue culture has been shown by several studies (Gago et al., 2010a, 2011, 2014; Yousefi and Razavi, 2017; Nezami-Alanagh et al., 2018).

Gago et al. (2010a) reported that neuro-fuzzy logic is a reliable and accurate model for studying *in vitro* rooting and acclimatization of *Vitis vinifera*. In another study, Gago et al. (2011) used the neuro-fuzzy logic to model the media formulation and reported that the neuro-fuzzy logic is a powerful model for modeling the effect of macronutrients on shoot proliferation of apricot. Alanagh et al. (2014) successfully used neuro-fuzzy logic to model the media formulation for shoot proliferation of *Prunus* rootstock.

Sensitivity analysis showed that 2,4-D and BAP have the maximum effect on embryogenesis, respectively, and also illustrated that fructose has little influence on this parameter. Previous studies have indicated that chrysanthemum embryogenesis response changes are caused by the type and concentration of PGRs (May and Trigiano, 1991; Tanaka et al., 2000; da Silva, 2003; Xu et al., 2012; Naing et al., 2013). da Silva et al. (2003) reported that the among auxins that tested, 2,4-D had the best response for somatic embryogenesis of chrysanthemum. Also, Mandal and Datta (2005) indicated that better results was achieved by using 2,4-D rather than other auxins. A similar result has been showed by Tanaka et al. (2000). 2,4-D as one of the most important synthetic auxins is of high paramount in *in vitro* culture, callus induction, and embryogenic cell systems in tissue culture (Zhao, 2008; Hesami and Daneshvar, 2018; Hesami et al., 2018). In addition, the significant effect of 2,4-D was reported on the physiological and molecular process of callus by adjusting the endogenous IAA metabolism, promoting specific proteins, and regulating the DNA methylation (Zhao, 2008).

The results of our study showed that the auxin/cytokinin (2,4-D/BAP) balance was necessary to obtain somatic embryogenesis. Also, the maximum embryogenesis was observed in 1.5 mg/L 2,4-D along with 1.5 mg/L BAP. As the same as our result, Naing et al. (2013) reported that the combination of 2.0 mg/L 2,4-D and 2.0 mg/L BA resulted in 100% embryogenesis in chrysanthemum. It is well-known that developing events in *in vitro* culture significantly depend on the ratio between cytokinins and auxins (Zhao, 2008; Hesami et al., 2018). According to the previous research, the external auxins during embryogenesis could be able to express some genes such as ARABIDOPSIS HISTIDINE KINASE4 (AHK4), YUCCA (YUC), and also auxin efflux carriers PINFORMED (PIN) that can be regulated by external cytokinins (Jones et al., 2010; Hesami et al., 2019a).

Type and concentration of carbohydrate have been reported to play critical roles in somatic embryogenesis (Fuentes et al., 2000; Blanc et al., 2002; Yancheva and Roichev, 2005). Although the majority of media applied sucrose as the standard carbohydrate source, many studies found that the beneficial effects of glucose on organogenesis (Fuentes et al., 2000; Blanc et al., 2002; Yancheva and Roichev, 2005; Yadollahi et al., 2011). Our results showed that the monosaccharides (fructose and glucose) and the disaccharide (sucrose) had different responses on callus formation and somatic embryogenesis. Although glucose and fructose were more effective at a higher concentration for promoting somatic embryogenesis, monosaccharides were less effective than disaccharides at the lower concentration (3%). However, there are some reports that have indicated the inhibitory effects of a high concentration of sucrose on somatic embryogenesis of different tissue derived from different species such as feijoa (Canhoto and Cruz, 1994), immature cotyledons of cassava (Konan et al., 1994), and petiole of *Medicago sativa* (Meijer and Brown, 1987). This inhibitory effect of the high level of sucrose is due to the increase of phenolics components in tissues that finally leads to cell death in the embryo (Kumar et al., 2015). According to our results, the high levels of somatic embryogenesis were obtained by 6% glucose. Similar to our results, da Silva (2004) demonstrated that glucose at 6% concentration was more efficient than sucrose for somatic embryogenesis of chrysanthemum. Also, our results showed that the maximum number of somatic embryo was achieved in the medium with 6% glucose. Similarly, Cunha and Ferreira (1999) demonstrated that the highest somatic embryogenesis (71.4%) of flax was obtained from 4% glucose and 4% fructose and the maximum number of the embryo was achieved in 4% glucose. Since glucose has the ability to oxidize phenolic components, it has a high potential for controlling the tissue browning (Abrie and Staden, 2001; Aydin et al., 2004; Kumar et al., 2015).

It is well-documented that the light quality could be able to regulate differentiation, growth, and morphogenesis of plant cell, tissue, and organ cultures (Reuveni and Evenor, 2007). Generally, the source of the light in plant tissue culture is provided by incandescent, high-pressure sodium, metal halide, and fluorescent lamps. However, some of these lamps exert a negative impact on plant growth due to the emission of non-essential wavelengths (Kim et al., 2004). Light-emitting diode (LED) such as the new light sources in plant tissue culture has several advantages such as provide relatively cool emitting surfaces, wavelength specificity and easy to detect their spectral composition, more durable, and much smaller in size (Chen et al., 2016). There various wavelengths provided by LED light can be matched to plant photoreceptors to influence metabolic and morphology composition of plants as well as providing optimal production (Bourget, 2008; Massa et al., 2008). Based on the merits discussed before, LED light sources are become more prominent for commercial *in vitro* cultivation of plants especially Phalaenopsis, chrysanthemum, Lilium, Cymbidium, birch, maize, potato, grape, strawberry, banana, and cotton (Sæb et al., 1995; Nhut et al., 2003; Li et al., 2010; Chen et al., 2016). One of the most important parameters that can exert a profound influence on the phenomics of the plants such as controlling growth and development and activating or deactivating physiological reactions is the integration, quality, duration and intensity of various visible light bands such as red-, infrared-, blue- and ultraviolet-light (Sæb et al., 1995; Briggs and Olney, 2001; Nhut et al., 2003; Li et al., 2010; Chen et al., 2016). Overall, several studies showed the successfulness of using LED light over fluorescent lamps in growing plants. There are several studies that have reported the significant effect of the light quality on somatic embryogenesis of different crops such as several pine species (Merkle et al., 2006), carrot (Michler and Lineberger, 1987), quince (D'onofrio et al., 1998), and China rose (Chen et al., 2014). Some of those studies indicated the effectiveness of using red light on increasing somatic embryogenesis frequency that probably related to the photo-equilibrium capability (D'onofrio et al., 1998; Chen et al., 2014). According to our study, the higher embryogenesis frequency was achieved by using red light as a light source in comparison with other light sources such as blue and white lights or darkness. Therefore, our results confirmed the successful use of red light in somatic embryo production.

The same as our result, Chen et al. (2014) demonstrated that red light had a better impact on somatic embryogenesis of China Rose compared with white color or darkness. Also, they reported that darkness was better than white color for embryogenesis. Red light exposure can exert a positive impact at two ways; 1- provide the maximum activation of phytochrome, 2- avoid the consequent and involvement negative impact of blue-absorbing photoreceptors (D'onofrio et al., 1998). In our study, the lowest somatic embryogenesis was achieved under blue light. Similarity to our results, (D'onofrio et al., 1998) reported that the minimum embryogenesis of quince was obtained from blue light. The negative effects of blue light may be due to the presence of low photo-equilibrium contents (D'onofrio et al., 1998; Chen et al., 2014).

## Conclusion

*In vitro* organogenesis is a multi-variable procedure that many factors such as PGRs, carbohydrate sources, medium composition, light, and temperature can affect its efficiency. Optimizing these conditions is necessary for being successful in plant tissue culture. Recently, there are various computational models proposed for solving the challenges exist in plant science areas, especially in plant tissue culture. Some of these models may be utilized in different studies in order to best predict the dependent variables. Our results showed that the ANFIS model is an efficient method for modeling and predicting complex systems such as somatic embryogenesis. Also, NSGAII allowed answers the questions on the optimal factors combination for achieving the most suitable results for the parameters studied. Generally, the hybrid ANFIS-NSGAII can be recognized as a powerful computational tool for modeling and optimizing in genetic engineering. Since this article was focused on the application of ANFIS model as one of the artificial intelligence models in plant tissue culture, it would be recommended to make a comparison with other artificial intelligence models such as MLP, SVR, and GRNN in further studies.

## Data Availability

No datasets were generated or analyzed for this study.

## Author Contributions

MH performing the experiments, modeling, summing up, and writing the manuscript. RN and MT designing and leading the experiments and revising the manuscript. MY-N revising the manuscript.

### Conflict of Interest Statement

The authors declare that the research was conducted in the absence of any commercial or financial relationships that could be construed as a potential conflict of interest.

## References

[B1] Abrie A. Staden J. V. (2001). Micropropagation of the endangered *Aloe polyphylla*. Plant Growth Regulat. 33, 19–23. 10.1023/A:1010725901900

[B2] Alanagh E. N. Garoosi G.-A. Haddad R. Maleki S. Landín M. Gallego P. P. (2014). Design of tissue culture media for efficient *Prunus* rootstock micropropagation using artificial intelligence models. Plant Cell Tissue Organ Cult. 117, 349–359. 10.1007/s11240-014-0444-1

[B3] Arab M. M. Yadollahi A. Eftekhari M. Ahmadi H. Akbari M. Khorami S. S. (2018). Modeling and optimizing a new culture medium for *in vitro* rooting of G × N15 prunus rootstock using artificial neural network-genetic algorithm. Sci. Report. 8:e9977 10.1038/s41598-018-27858-4 PMC6028477 29967468

[B4] Arab M. M. Yadollahi A. Shojaeiyan A. Ahmadi H. (2016). Artificial neural network genetic algorithm as powerful tool to predict and optimize *in vitro* proliferation mineral medium for G × N15 rootstock. Front. Plant Sci. 7:e1526. 10.3389/fpls.2016.01526 27807436 PMC5069296

[B5] Aydin Y. Ipekci Z. Talas-Ogra,ş T. Zehir A. Bajrovic K. Gozukirmizi N. (2004). High frequency somatic embryogenesis in cotton. Biol. Plant. 48, 491–495. 10.1023/B:BIOP.0000047142.07987.e1

[B6] Blanc G. Lardet L. Martin A. Jacob J.-L. Carron M.-P. (2002). Differential carbohydrate metabolism conducts morphogenesis in embryogenic callus of *Hevea brasiliensis* (Mull. *Arg*.). J. Exp. Bot. 53, 1453–1462. 10.1093/jexbot/53.373.1453 12021293

[B7] Bourget C. M. (2008). An introduction to light-emitting diodes. HortScience 43, 1944–1946. 10.21273/HORTSCI.43.7.1944

[B8] Bozorg-Haddad O. Azarnivand A. Hosseini-Moghari S.-M. Loáiciga H. A. (2016). Development of a comparative multiple criteria framework for ranking pareto optimal solutions of a multiobjective reservoir operation problem. J. Irrigat. Drainage Eng. 142:e04016019 10.1061/(ASCE)IR.1943-4774.0001028

[B9] Briggs W. R. Olney M. A. (2001). Photoreceptors in plant photomorphogenesis to date. Five phytochromes, two cryptochromes, one phototropin, and one superchrome. Plant Physiol. 125, 85–88. 10.1104/pp.125.1.85 11154303 PMC1539332

[B10] Canhoto J. M. Cruz G. S. (1994). Improvement of somatic embryogenesis in *Feijoa sellowiana* berg (*Myrtaceae*) by manipulation of culture media composition. In Vitro Cell. Dev. Biol. Plant 30, 21–25. 10.1007/BF02632115

[B11] Chen C.-C. Agrawal D. C. Lee M.-R. Lee R.-J. Kuo C.-L. Wu C.-R.. (2016). Influence of LED light spectra on *in vitro* somatic embryogenesis and LC–MS analysis of chlorogenic acid and rutin in *Peucedanum japonicum* Thunb.: a medicinal herb. Bot. Stud. 57:e9. 10.1186/s40529-016-0124-z 28597418 PMC5430566

[B12] Chen J.-R. Wu L. Hu B.-W. Yi X. Liu R. Deng Z.-N. (2014). The influence of plant growth regulators and light quality on somatic embryogenesis in China rose (*Rosa chinensis* Jacq.). J. Plant Growth Regulat. 33, 295–304. 10.1007/s00344-013-9371-3

[B13] Cunha A. Ferreira M. F. (1999). Influence of medium parameters on somatic embryogenesis from hypocotyl explants of flax (*Linum usitatissimum* L.): effect of carbon source, total inorganic nitrogen and balance between ionic forms and interaction between calcium and zeatin. J. Plant Physiol. 213, 591–597. 10.1016/S0176-1617(99)80059-5

[B14] da Silva J. A. (2004). The effect of carbon source on *in vitro* organogenesis of chrysanthemum thin cell layers. Bragantia 63, 165–177. 10.1590/S0006-87052004000200002

[B15] da Silva T. J. A. (2003). Chrysanthemum: advances in tissue culture, cryopreservation, postharvest technology, genetics and transgenic biotechnology. Biotechnol. Adv. 21, 715–766. 10.1016/S0734-9750(03)00117-4 14563477

[B16] da Silva T. J. A. Kulus D. (2014). Chrysanthemum biotechnology: discoveries from the recent literature. Folia Horticult. 26, 67–77. 10.2478/fhort-2014-0007

[B17] da Silva T. J. A. Nhut D. T. Tanaka M. Fukai S. (2003). The effect of antibiotics on the *in vitro* growth response of chrysanthemum and tobacco stem transverse thin cell layers (tTCLs). Sci. Horticult. 97, 397–410. 10.1016/S0304-4238(02)00219-4

[B18] D'onofrio C. Morini S. Bellocchi G. (1998). Effect of light quality on somatic embryogenesis of quince leaves. Plant Cell Tissue Organ Cult. 53, 91–98. 10.1023/A:1006059615088

[B19] Elhiti M. Stasolla C. Wang A. (2013). Molecular regulation of plant somatic embryogenesis. In Vitro Cell. Dev. Biol. Plant 49, 631–642. 10.1007/s11627-013-9547-3

[B20] Farokhnia A. Morid S. Byun H.-R. (2011). Application of global SST and SLP data for drought forecasting on Tehran plain using data mining and ANFIS techniques. Theoret.Appl. Climatol. 104, 71–81. 10.1007/s00704-010-0317-4

[B21] Ferreira L. T. De Araújo Silva M. M. Ulisses C. Camara T. R. Willadino L. J. P. C. Tissue (2017). Using LED lighting in somatic embryogenesis and micropropagation of an elite sugarcane variety and its effect on redox metabolism during acclimatization. Plant Cell Tissue Organ Cult. 128, 211–221. 10.1007/s11240-016-1101-7

[B22] Fuentes S. R. Calheiros M. B. Manetti-Filho J. Vieira L. G. (2000). The effects of silver nitrate and different carbohydrate sources on somatic embryogenesis in *Coffea canephora*. Plant Cell Tissue Organ Cult. 60, 5–13. 10.1023/A:1006474324652

[B23] Gago J. Landín M. Gallego P. P. (2010a). A neurofuzzy logic approach for modeling plant processes: a practical case of *in vitro* direct rooting and acclimatization of *Vitis vinifera* L. Plant Sci. 179, 241–249. 10.1016/j.plantsci.2010.05.009

[B24] Gago J. Martínez-Núñez L. Landín M. Flexas J. Gallego P. P. (2014). Modeling the effects of light and sucrose on *in vitro* propagated plants: a multiscale system analysis using artificial intelligence technology. PLoS ONE 9:e85989. 10.1371/journal.pone.0085989 24465829 PMC3896442

[B25] Gago J. Martínez-Núñez L. Landín M. Gallego P. (2010b). Artificial neural networks as an alternative to the traditional statistical methodology in plant research. J. Plant Physiol. 167, 23–27. 10.1016/j.jplph.2009.07.007 19716625

[B26] Gago J. Pérez-Tornero O. Landín M. Burgos L. Gallego P. P. (2011). Improving knowledge of plant tissue culture and media formulation by neurofuzzy logic: a practical case of data mining using apricot databases. J. Plant Physiol. 168, 1858–1865. 10.1016/j.jplph.2011.04.008 21676490

[B27] Hesami M. Daneshvar M. H. (2018). *In vitro* adventitious shoot regeneration through direct and indirect organogenesis from seedling-derived hypocotyl segments of *Ficus religiosa* L.: an important medicinal plant. HortScience 53, 55–61. 10.21273/HORTSCI12637-17

[B28] Hesami M. Daneshvar M. H. Yoosefzadeh-Najafabadi M. (2019a). An efficient *in vitro* shoot regeneration through direct organogenesis from seedling-derived petiole and leaf segments and acclimatization of *Ficus religiosa*. J Forestry Res. 30, 807–815. 10.1007/s11676-018-0647-0

[B29] Hesami M. Daneshvar M. H. Yoosefzadeh-Najafabadi M. Alizadeh M. (2018). Effect of plant growth regulators on indirect shoot organogenesis of *Ficus religiosa* through seedling derived petiole segments. J Genet. Eng. Biotechnol. 16, 175–180. 10.1016/j.jgeb.2017.11.001 30647720 PMC6296569

[B30] Hesami M. Naderi R. Tohidfar M. (2019b). Modeling and optimizing *in vitro* sterilization of chrysanthemum via multilayer perceptron-non-dominated sorting genetic algorithm-II (MLP-NSGAII). Front. Plant Sci. 10:282. 10.3389/fpls.2019.00282 30923529 PMC6426794

[B31] Hesami M. Naderi R. Yoosefzadeh-Najafabadi M. Rahmati M. (2017). Data-driven modeling in plant tissue culture. J. Appl. Environ. Biol. Sci. 7, 37–44.

[B32] Hosseini-Moghari S.-M. Araghinejad S. Azarnivand A. (2017). Drought forecasting using data-driven methods and an evolutionary algorithm. Model. Earth Syst. Environ. 3, 1675–1689. 10.1007/s40808-017-0385-x

[B33] Jamshidi S. Yadollahi A. Ahmadi H. Arab M. Eftekhari M. (2016). Predicting *in vitro* culture medium macro-nutrients composition for pear rootstocks using regression analysis and neural network models. Front. Plant Sci. 7:e274. 10.3389/fpls.2016.00274 27066013 PMC4809900

[B34] Jones B. Gunnerås S. A. Petersson S. V. Tarkowski P. Graham N. May S.. (2010). Cytokinin regulation of auxin synthesis in Arabidopsis involves a homeostatic feedback loop regulated via auxin and cytokinin signal transduction. Plant Cell 22, 2956–2969. 10.1105/tpc.110.074856 20823193 PMC2965550

[B35] Kendal D. Hauser C. E. Garrard G. E. Jellinek S. Giljohann K. M. Moore J. L. (2013). Quantifying plant colour and colour difference as perceived by humans using digital images. PLoS ONE 8:e72296. 10.1371/journal.pone.0072296 23977275 PMC3748102

[B36] Kim S.-J. Hahn E.-J. Heo J.-W. Paek K.-Y. (2004). Effects of LEDs on net photosynthetic rate, growth and leaf stomata of chrysanthemum plantlets *in vitro*. Scient. Horticult. 101, 143–151. 10.1016/j.scienta.2003.10.003

[B37] Konan N. Sangwan R. Sangwan B. (1994). Somatic embryogenesis from cultured mature cotyledons of cassava (*Manihot esculenta* Crantz). Plant Cell Tissue Organ Cult. 37, 91–102. 10.1007/BF00043602

[B38] Kumar G. P. Subiramani S. Govindarajan S. Sadasivam V. Manickam V. Mogilicherla K.. (2015). Evaluation of different carbon sources for high frequency callus culture with reduced phenolic secretion in cotton (*Gossypium hirsutum* L.) cv. SVPR-2. Biotechnol. Rep. 7, 72–80. 10.1016/j.btre.2015.05.005 28626717 PMC5466046

[B39] Łapa K. Cpałka K. Rutkowski L. (2018). New aspects of interpretability of fuzzy systems for nonlinear modeling, in Advances in Data Analysis With Computational Intelligence Methods eds Gawȩda A. Kacprzyk J. Rutkowski L. Yen G. (Cham: Springer), 225–264. 10.1007/978-3-319-67946-4_9

[B40] Li H. Wang J. Du H. Karimi H. R. (2018). Adaptive sliding mode control for Takagi–Sugeno fuzzy systems and its applications. IEEE Transact. Fuzzy Syst. 26, 531–542. 10.1109/TFUZZ.2017.2686357

[B41] Li H. Xu Z. Tang C. (2010). Effect of light-emitting diodes on growth and morphogenesis of upland cotton (*Gossypium hirsutum* L.) plantlets *in vitro*. Plant Cell Tissue Organ Cult. 103, 155–163. 10.1007/s11240-010-9763-z

[B42] Mandal A. Datta S. (2005). Direct somatic embryogenesis and plant regeneration from ray florets of chrysanthemum. Biol. Plant. 49, 29–33. 10.1007/s10535-005-0033-6

[B43] Massa G. D. Kim H.-H. Wheeler R. M. Mitchell C. A. (2008). Plant productivity in response to LED lighting. HortScience 43, 1951–1956. 10.21273/HORTSCI.43.7.1951

[B44] May R. Trigiano R. (1991). Somatic embryogenesis and plant regeneration from leaves of *Dendranthema grandiflora*. J. Am. Soc. Horticult. Sci. 116, 366–371. 10.21273/JASHS.116.2.366

[B45] Meijer E. G. Brown D. C. (1987). Role of exogenous reduced nitrogen and sucrose in rapid high frequency somatic embryogenesis in *Medicago sativa*. Plant Cell Tissue Organ Cult. 10, 11–19. 10.1007/BF00037492

[B46] Merkle S. A. Montello P. M. Xia X. Upchurch B. L. Smith D. R. (2006). Light quality treatments enhance somatic seedling production in three southern pine species. Tree Physiol. 26, 187–194. 10.1093/treephys/26.2.187 16356915

[B47] Michler C. H. Lineberger R. D. (1987). Effects of light on somatic embryo development and abscisic levels in carrot suspension cultures. Plant Cell Tissue Organ Cult. 11, 189–207. 10.1007/BF00040425

[B48] Murashige T. Skoog F. (1962). A revised medium for rapid growth and bio assays with tobacco tissue cultures. Physiol. Plant. 15, 473–497. 10.1111/j.1399-3054.1962.tb08052.x

[B49] Naing A. H. Kim C. K. Yun B. J. Jin J. Y. Lim K. B. (2013). Primary and secondary somatic embryogenesis in *Chrysanthemum* cv. Euro. Plant Cell Tissue Organ Cult. 112, 361–368. 10.1007/s11240-012-0243-5

[B50] Nezami-Alanagh E. Garoosi G.-A. Landin M. Gallego P. P. (2018). Combining DOE with neurofuzzy logic for healthy mineral nutrition of pistachio rootstocks *in vitro* culture. Front. Plant Sci. 9:e1474. 10.3389/fpls.2018.01474 30374362 PMC6196285

[B51] Nezami-Alanagh E. Garoosi G.-A. Maleki S. Landín M. Gallego P. P. (2017). Predicting optimal *in vitro* culture medium for *Pistacia vera* micropropagation using neural networks models. Plant Cell Tissue Organ Cult. 129, 19–33. 10.1007/s11240-016-1152-9

[B52] Nhut D. T. Huy N. P. Tai N. T. Nam N. B. Luan V. Q. Hien V. T.. (2015). Light-emitting diodes and their potential in callus growth, plantlet development and saponin accumulation during somatic embryogenesis of *Panax vietnamensis* Ha et Grushv. Biotechnol. Biotechnol. Equip. 29, 299–308. 10.1080/13102818.2014.1000210 26019644 PMC4433904

[B53] Nhut D. T. Takamura T. Watanabe H. Okamoto K. Tanaka M. (2003). Responses of strawberry plantlets cultured *in vitro* under superbright red and blue light-emitting diodes (LEDs). Plant Cell Tissue Organ Cult. 73, 43–52. 10.1023/A:1022638508007

[B54] Niazian M. Sadat-Noori S. A. Abdipour M. Tohidfar M. Mortazavian S. M. M. (2018). Image processing and artificial neural network-based models to measure and predict physical properties of embryogenic callus and number of somatic embryos in ajowan (*Trachyspermum ammi* (L.) Sprague). In Vitro Cell Dev. Biol. Plant 54, 54–68. 10.1007/s11627-017-9877-7

[B55] Noda N. Yoshioka S. Kishimoto S. Nakayama M. Douzono M. Tanaka Y.. (2017). Generation of blue chrysanthemums by anthocyanin B-ring hydroxylation and glucosylation and its coloration mechanism. Sci. Adv. 3:e1602785. 10.1126/sciadv.1602785 28782017 PMC5529055

[B56] Pavingerová D. Dostál J. Bískov,á R. Benetka V. (1994). Somatic embryogenesis and *Agrobacterium*-mediated transformation of chrysanthemum. Plant Sci. 97, 95–101. 10.1016/0168-9452(94)90111-2

[B57] Prakash O. Mehrotra S. Krishna A. Mishra B. N. (2010). A neural network approach for the prediction of *in vitro* culture parameters for maximum biomass yields in hairy root cultures. J. Theoret. Biol. 265, 579–585. 10.1016/j.jtbi.2010.05.020 20561985

[B58] Reuveni M. Evenor D. (2007). On the effect of light on shoot regeneration in petunia. Plant Cell Tissue Organ Cult. 89, 49–54. 10.1007/s11240-007-9215-6

[B59] Sæb,ø A. Krekling T. Appelgren M. (1995). Light quality affects photosynthesis and leaf anatomy of birch plantlets *in vitro*. Plant Cell Tissue Organ Cult. 41, 177–185. 10.1007/BF00051588

[B60] Shinoyama H. Nomura Y. Tsuchiya T. Kazuma T. (2004). A simple and efficient method for somatic embryogenesis and plant regeneration from leaves of chrysanthemum [*Dendranthema* × *grandiflorum* (Ramat.) Kitamura]. Pant Biotechnol. 21, 25–33. 10.5511/plantbiotechnology.21.25

[B61] Tanaka K. Kanno Y. Kudo S. Suzuki M. (2000). Somatic embryogenesis and plant regeneration in chrysanthemum (*Dendranthema grandiflorum* (Ramat.) Kitamura). Plant Cell Rep. 19, 946–953. 10.1007/s002990000225 30754837

[B62] Wang Y. Shen Y. Zhang X. Cui G. Sun J. (2018). An Improved Non-dominated Sorting Genetic Algorithm-II (INSGA-II) applied to the design of DNA codewords. Mathemat. Comput. Simulat. 151, 131–139. 10.1016/j.matcom.2018.03.011

[B63] Xu P. Zhang Z. Wang B. Xia X. Jia J. (2012). Somatic embryogenesis and plant regeneration in chrysanthemum (Yuukou). Plant Cell Tissue Organ Cult. 111, 393–397. 10.1007/s11240-012-0201-2

[B64] Yadollahi A. Abdollahi M. Moieni A. Danaee M. (2011). Effects of carbon source, polyethylene glycol and abscisic acid on secondary embryo induction and maturation in rapeseed (*Brassica napus* L.) microspore-derived embryos. Acta Physiol. Plant. 33, 1905–1912. 10.1007/s11738-011-0738-4

[B65] Yancheva S. Roichev V. (2005). Carbohydrate source can influence the efficiency of somatic embryogenesis in seedless grapes (*Vitis vinifera* L.). Biotechnol. Biotechnol. Equip. 19, 62–66. 10.1080/13102818.2005.10817192

[B66] Yousefi A. Razavi S. M. (2017). Modeling of glucose release from native and modified wheat starch gels during *in vitro* gastrointestinal digestion using artificial intelligence methods. Int. J. Biol. Macromol. 97, 752–760. 10.1016/j.ijbiomac.2017.01.082 28111297

[B67] Zhao Y. (2008). The role of local biosynthesis of auxin and cytokinin in plant development. Curr. Opin. Plant Biol. 11, 16–22. 10.1016/j.pbi.2007.10.008 18409210

