# Supplementary material for: Application of Adaptive Neuro-Fuzzy Inference System-Non-dominated Sorting Genetic Algorithm-II (ANFIS-NSGAII) for Modeling and Optimizing Somatic Embryogenesis of Chrysanthemum
Source: Front Plant Sci. 2019 Jul 5;10:869. doi: 10.3389/fpls.2019.00869 (PMC6624437; doi:10.3389/fpls.2019.00869)
Supplement: Supplementary file 1 [file Table_1.DOCX]

| **Table S1.** Effects of 2,4-D, BAP, glucose (GLU), sucrose (SUC), fructose (FRU), and light (R,G,B) on callogenesis frequency (CF), embryogenesis frequency (EF), and number of somatic embryo (NSE) of chrysanthemum. | | | | | | | | | | | | |
| --- | --- | --- | --- | --- | --- | --- | --- | --- | --- | --- | --- | --- |
| NO. | Inputs | | | | | | | |  | Outputs | | |
|  | 2,4-D (mg/l) | BAP (mg/l) | GLU (g/l) | SUC (g/l) | FRU (g/l) | R | G | B |  | CF (%) | EF (%) | NSE |
| 1 | 0.5 | 0.5 | 30 | 0 | 0 | 0 | 0 | 0 |  | 0.00±0.00 | 0.00±0.00 | 0.00±0.00 |
| 2 | 0.5 | 2.5 | 30 | 0 | 0 | 0 | 0 | 0 |  | 2.22±2.22 | 0.00±0.00 | 0.00±0.00 |
| 3 | 0.5 | 1.5 | 30 | 0 | 0 | 0 | 0 | 0 |  | 6.67±3.85 | 0.00±0.00 | 0.00±0.00 |
| 4 | 2.5 | 0.5 | 30 | 0 | 0 | 0 | 0 | 0 |  | 22.22±5.88 | 0.00±0.00 | 0.00±0.00 |
| 5 | 2.5 | 2.5 | 30 | 0 | 0 | 0 | 0 | 0 |  | 95.56±2.22 | 68.89±11.76 | 4.87±1.02 |
| 6 | 2.5 | 1.5 | 30 | 0 | 0 | 0 | 0 | 0 |  | 77.78±8.01 | 48.89±8.01 | 3.73±0.73 |
| 7 | 1.5 | 0.5 | 30 | 0 | 0 | 0 | 0 | 0 |  | 68.89±5.88 | 0.00±0.00 | 0.00±0.00 |
| 8 | 1.5 | 2.5 | 30 | 0 | 0 | 0 | 0 | 0 |  | 97.78±2.22 | 77.78±5.88 | 4.00±0.21 |
| 9 | 1.5 | 1.5 | 30 | 0 | 0 | 0 | 0 | 0 |  | 100.00±0.00 | 93.33±3.85 | 6.63±0.50 |
| 10 | 0.5 | 0.5 | 60 | 0 | 0 | 0 | 0 | 0 |  | 0.00±0.00 | 0.00±0.00 | 0.00±0.00 |
| 11 | 0.5 | 2.5 | 60 | 0 | 0 | 0 | 0 | 0 |  | 0.00±0.00 | 0.00±0.00 | 0.00±0.00 |
| 12 | 0.5 | 1.5 | 60 | 0 | 0 | 0 | 0 | 0 |  | 4.44±2.22 | 0.00±0.00 | 0.00±0.00 |
| 13 | 2.5 | 0.5 | 60 | 0 | 0 | 0 | 0 | 0 |  | 24.44±5.88 | 0.00±0.00 | 0.00±0.00 |
| 14 | 2.5 | 2.5 | 60 | 0 | 0 | 0 | 0 | 0 |  | 100.00±0.00 | 75.56±5.88 | 5.17±0.99 |
| 15 | 2.5 | 1.5 | 60 | 0 | 0 | 0 | 0 | 0 |  | 84.44±5.88 | 55.56±4.44 | 4.03±0.75 |
| 16 | 1.5 | 0.5 | 60 | 0 | 0 | 0 | 0 | 0 |  | 73.33±3.85 | 0.00±0.00 | 0.00±0.00 |
| 17 | 1.5 | 2.5 | 60 | 0 | 0 | 0 | 0 | 0 |  | 100.00±0.00 | 82.22±2.22 | 4.43±0.24 |
| 18 | 1.5 | 1.5 | 60 | 0 | 0 | 0 | 0 | 0 |  | 100.00±0.00 | 100.00±0.00 | 10.47±0.52 |
| 19 | 0.5 | 0.5 | 0 | 60 | 0 | 0 | 0 | 0 |  | 0.00±0.00 | 0.00±0.00 | 0.00±0.00 |
| 20 | 0.5 | 2.5 | 0 | 60 | 0 | 0 | 0 | 0 |  | 0.00±0.00 | 0.00±0.00 | 0.00±0.00 |
| 21 | 0.5 | 1.5 | 0 | 60 | 0 | 0 | 0 | 0 |  | 4.44±4.44 | 0.00±0.00 | 0.00±0.00 |
| 22 | 2.5 | 0.5 | 0 | 60 | 0 | 0 | 0 | 0 |  | 11.11±4.44 | 0.00±0.00 | 0.00±0.00 |
| 23 | 2.5 | 2.5 | 0 | 60 | 0 | 0 | 0 | 0 |  | 66.67±6.67 | 57.78±8.01 | 3.67±0.46 |
| 24 | 2.5 | 1.5 | 0 | 60 | 0 | 0 | 0 | 0 |  | 62.22±2.22 | 42.22±4.44 | 2.80±0.21 |
| 25 | 1.5 | 0.5 | 0 | 60 | 0 | 0 | 0 | 0 |  | 51.11±2.22 | 0.00±0.00 | 0.00±0.00 |
| 26 | 1.5 | 2.5 | 0 | 60 | 0 | 0 | 0 | 0 |  | 64.44±8.01 | 64.44±8.89 | 3.17±0.15 |
| 27 | 1.5 | 1.5 | 0 | 60 | 0 | 0 | 0 | 0 |  | 68.89±8.89 | 64.44±8.01 | 6.13±0.32 |
| 28 | 0.5 | 0.5 | 0 | 30 | 0 | 0 | 0 | 0 |  | 0.00±0.00 | 0.00±0.00 | 0.00±0.00 |
| 29 | 0.5 | 2.5 | 0 | 30 | 0 | 0 | 0 | 0 |  | 0.00±0.00 | 0.00±0.00 | 0.00±0.00 |
| 30 | 0.5 | 1.5 | 0 | 30 | 0 | 0 | 0 | 0 |  | 2.22±2.22 | 0.00±0.00 | 0.00±0.00 |
| 31 | 2.5 | 0.5 | 0 | 30 | 0 | 0 | 0 | 0 |  | 17.78±5.88 | 0.00±0.00 | 0.00±0.00 |
| 32 | 2.5 | 2.5 | 0 | 30 | 0 | 0 | 0 | 0 |  | 68.89±5.88 | 62.22±2.22 | 4.70±0.55 |
| 33 | 2.5 | 1.5 | 0 | 30 | 0 | 0 | 0 | 0 |  | 80.00±6.67 | 51.11±2.22 | 3.33±0.22 |
| 34 | 1.5 | 0.5 | 0 | 30 | 0 | 0 | 0 | 0 |  | 68.89±2.22 | 0.00±0.00 | 0.00±0.00 |
| 35 | 1.5 | 2.5 | 0 | 30 | 0 | 0 | 0 | 0 |  | 91.11±4.44 | 75.56±4.44 | 4.20±0.26 |
| 36 | 1.5 | 1.5 | 0 | 30 | 0 | 0 | 0 | 0 |  | 95.56±2.22 | 93.33±3.85 | 9.73±0.38 |
| 37 | 0.5 | 0.5 | 0 | 0 | 30 | 0 | 0 | 0 |  | 0.00±0.00 | 0.00±0.00 | 0.00±0.00 |
| 38 | 0.5 | 2.5 | 0 | 0 | 30 | 0 | 0 | 0 |  | 0.00±0.00 | 0.00±0.00 | 0.00±0.00 |
| 39 | 0.5 | 1.5 | 0 | 0 | 30 | 0 | 0 | 0 |  | 0.00±0.00 | 0.00±0.00 | 0.00±0.00 |
| 40 | 2.5 | 0.5 | 0 | 0 | 30 | 0 | 0 | 0 |  | 4.44±2.22 | 0.00±0.00 | 0.00±0.00 |
| 41 | 2.5 | 2.5 | 0 | 0 | 30 | 0 | 0 | 0 |  | 51.11±4.44 | 53.33±6.67 | 3.27±0.46 |
| 42 | 2.5 | 1.5 | 0 | 0 | 30 | 0 | 0 | 0 |  | 46.67±6.67 | 37.78±2.22 | 2.53±0.07 |
| 43 | 1.5 | 0.5 | 0 | 0 | 30 | 0 | 0 | 0 |  | 44.44±2.22 | 0.00±0.00 | 0.00±0.00 |
| 44 | 1.5 | 2.5 | 0 | 0 | 30 | 0 | 0 | 0 |  | 60.00±3.85 | 48.89±9.69 | 2.70±0.23 |
| 45 | 1.5 | 1.5 | 0 | 0 | 30 | 0 | 0 | 0 |  | 55.56±8.89 | 46.67±10.18 | 5.23±0.70 |
| 46 | 0.5 | 0.5 | 0 | 0 | 60 | 0 | 0 | 0 |  | 0.00±0.00 | 0.00±0.00 | 0.00±0.00 |
| 47 | 0.5 | 2.5 | 0 | 0 | 60 | 0 | 0 | 0 |  | 0.00±0.00 | 0.00±0.00 | 0.00±0.00 |
| 48 | 0.5 | 1.5 | 0 | 0 | 60 | 0 | 0 | 0 |  | 0.00±0.00 | 0.00±0.00 | 0.00±0.00 |
| 49 | 2.5 | 0.5 | 0 | 0 | 60 | 0 | 0 | 0 |  | 15.56±4.44 | 0.00±0.00 | 0.00±0.00 |
| 50 | 2.5 | 2.5 | 0 | 0 | 60 | 0 | 0 | 0 |  | 64.44±2.22 | 44.44±4.44 | 3.77±0.44 |
| 51 | 2.5 | 1.5 | 0 | 0 | 60 | 0 | 0 | 0 |  | 71.11±2.22 | 44.44±2.22 | 2.93±0.26 |
| 52 | 1.5 | 0.5 | 0 | 0 | 60 | 0 | 0 | 0 |  | 57.78±5.88 | 0.00±0.00 | 0.00±0.00 |
| 53 | 1.5 | 2.5 | 0 | 0 | 60 | 0 | 0 | 0 |  | 80.00±6.67 | 62.22±5.88 | 3.97±0.07 |
| 54 | 1.5 | 1.5 | 0 | 0 | 60 | 0 | 0 | 0 |  | 82.22±4.44 | 84.44±2.22 | 9.10±0.35 |
| 55 | 0.5 | 0.5 | 90 | 0 | 0 | 0 | 0 | 0 |  | 0.00±0.00 | 0.00±0.00 | 0.00±0.00 |
| 56 | 0.5 | 2.5 | 90 | 0 | 0 | 0 | 0 | 0 |  | 4.44±2.22 | 0.00±0.00 | 0.00±0.00 |
| 57 | 0.5 | 1.5 | 90 | 0 | 0 | 0 | 0 | 0 |  | 8.89±2.22 | 0.00±0.00 | 0.00±0.00 |
| 58 | 2.5 | 0.5 | 90 | 0 | 0 | 0 | 0 | 0 |  | 24.44±4.44 | 0.00±0.00 | 0.00±0.00 |
| 59 | 2.5 | 2.5 | 90 | 0 | 0 | 0 | 0 | 0 |  | 97.78±2.22 | 73.33±7.70 | 5.07±1.00 |
| 60 | 2.5 | 1.5 | 90 | 0 | 0 | 0 | 0 | 0 |  | 82.22±5.88 | 55.56±2.22 | 3.83±0.81 |
| 61 | 1.5 | 0.5 | 90 | 0 | 0 | 0 | 0 | 0 |  | 73.33±3.85 | 0.00±0.00 | 0.00±0.00 |
| 62 | 1.5 | 2.5 | 90 | 0 | 0 | 0 | 0 | 0 |  | 97.78±2.22 | 82.22±2.22 | 4.10±0.20 |
| 63 | 1.5 | 1.5 | 90 | 0 | 0 | 0 | 0 | 0 |  | 100.00±0.00 | 95.56±2.22 | 6.83±0.41 |
| 64 | 0.5 | 0.5 | 0 | 20 | 0 | 0 | 0 | 0 |  | 0.00±0.00 | 0.00±0.00 | 0.00±0.00 |
| 65 | 0.5 | 2.5 | 0 | 20 | 0 | 0 | 0 | 0 |  | 0.00±0.00 | 0.00±0.00 | 0.00±0.00 |
| 66 | 0.5 | 1.5 | 0 | 20 | 0 | 0 | 0 | 0 |  | 2.22±2.22 | 0.00±0.00 | 0.00±0.00 |
| 67 | 2.5 | 0.5 | 0 | 20 | 0 | 0 | 0 | 0 |  | 15.56±2.22 | 0.00±0.00 | 0.00±0.00 |
| 68 | 2.5 | 2.5 | 0 | 20 | 0 | 0 | 0 | 0 |  | 62.22±2.22 | 64.44±4.44 | 4.70±0.95 |
| 69 | 2.5 | 1.5 | 0 | 20 | 0 | 0 | 0 | 0 |  | 71.11±8.89 | 46.67±0.00 | 3.20±0.25 |
| 70 | 1.5 | 0.5 | 0 | 20 | 0 | 0 | 0 | 0 |  | 66.67±6.67 | 0.00±0.00 | 0.00±0.00 |
| 71 | 1.5 | 2.5 | 0 | 20 | 0 | 0 | 0 | 0 |  | 68.89±11.11 | 71.11±2.22 | 3.97±0.07 |
| 72 | 1.5 | 1.5 | 0 | 20 | 0 | 0 | 0 | 0 |  | 88.89±2.22 | 75.56±11.76 | 9.00±0.32 |
| 73 | 0.5 | 0.5 | 20 | 20 | 20 | 0 | 0 | 0 |  | 0.00±0.00 | 0.00±0.00 | 0.00±0.00 |
| 74 | 0.5 | 2.5 | 20 | 20 | 20 | 0 | 0 | 0 |  | 0.00±0.00 | 0.00±0.00 | 0.00±0.00 |
| 75 | 0.5 | 1.5 | 20 | 20 | 20 | 0 | 0 | 0 |  | 6.67±0.00 | 0.00±0.00 | 0.00±0.00 |
| 76 | 2.5 | 0.5 | 20 | 20 | 20 | 0 | 0 | 0 |  | 22.22±2.22 | 0.00±0.00 | 0.00±0.00 |
| 77 | 2.5 | 2.5 | 20 | 20 | 20 | 0 | 0 | 0 |  | 95.56±2.22 | 68.89±4.44 | 4.23±0.77 |
| 78 | 2.5 | 1.5 | 20 | 20 | 20 | 0 | 0 | 0 |  | 80.00±6.67 | 51.11±4.44 | 3.60±0.61 |
| 79 | 1.5 | 0.5 | 20 | 20 | 20 | 0 | 0 | 0 |  | 71.11±4.44 | 0.00±0.00 | 0.00±0.00 |
| 80 | 1.5 | 2.5 | 20 | 20 | 20 | 0 | 0 | 0 |  | 91.11±4.44 | 77.78±2.22 | 3.83±0.38 |
| 81 | 1.5 | 1.5 | 20 | 20 | 20 | 0 | 0 | 0 |  | 93.33±0.00 | 93.33±0.00 | 6.23±0.09 |
| 82 | 0.5 | 0.5 | 0 | 0 | 90 | 0 | 0 | 0 |  | 0.00±0.00 | 0.00±0.00 | 0.00±0.00 |
| 83 | 0.5 | 2.5 | 0 | 0 | 90 | 0 | 0 | 0 |  | 0.00±0.00 | 0.00±0.00 | 0.00±0.00 |
| 84 | 0.5 | 1.5 | 0 | 0 | 90 | 0 | 0 | 0 |  | 0.00±0.00 | 0.00±0.00 | 0.00±0.00 |
| 85 | 2.5 | 0.5 | 0 | 0 | 90 | 0 | 0 | 0 |  | 0.00±0.00 | 0.00±0.00 | 0.00±0.00 |
| 86 | 2.5 | 2.5 | 0 | 0 | 90 | 0 | 0 | 0 |  | 37.78±4.44 | 35.56±2.22 | 3.63±0.48 |
| 87 | 2.5 | 1.5 | 0 | 0 | 90 | 0 | 0 | 0 |  | 53.33±6.67 | 35.56±2.22 | 2.90±0.23 |
| 88 | 1.5 | 0.5 | 0 | 0 | 90 | 0 | 0 | 0 |  | 46.67±6.67 | 0.00±0.00 | 0.00±0.00 |
| 89 | 1.5 | 2.5 | 0 | 0 | 90 | 0 | 0 | 0 |  | 60.00±6.67 | 53.33±6.67 | 3.40±0.25 |
| 90 | 1.5 | 1.5 | 0 | 0 | 90 | 0 | 0 | 0 |  | 71.11±2.22 | 57.78±4.44 | 5.97±0.47 |
| 91 | 0.5 | 0.5 | 30 | 0 | 0 | 255 | 0 | 0 |  | 0.00±0.00 | 0.00±0.00 | 0.00±0.00 |
| 92 | 0.5 | 2.5 | 30 | 0 | 0 | 255 | 0 | 0 |  | 4.44±2.22 | 0.00±0.00 | 0.00±0.00 |
| 93 | 0.5 | 1.5 | 30 | 0 | 0 | 255 | 0 | 0 |  | 8.89±2.22 | 0.00±0.00 | 0.00±0.00 |
| 94 | 2.5 | 0.5 | 30 | 0 | 0 | 255 | 0 | 0 |  | 28.89±2.22 | 0.00±0.00 | 0.00±0.00 |
| 95 | 2.5 | 2.5 | 30 | 0 | 0 | 255 | 0 | 0 |  | 97.78±2.22 | 75.56±5.88 | 5.47±0.38 |
| 96 | 2.5 | 1.5 | 30 | 0 | 0 | 255 | 0 | 0 |  | 84.44±4.44 | 57.78±2.22 | 4.97±0.24 |
| 97 | 1.5 | 0.5 | 30 | 0 | 0 | 255 | 0 | 0 |  | 71.11±4.44 | 0.00±0.00 | 0.00±0.00 |
| 98 | 1.5 | 2.5 | 30 | 0 | 0 | 255 | 0 | 0 |  | 100.00±0.00 | 84.44±2.22 | 4.43±0.07 |
| 99 | 1.5 | 1.5 | 30 | 0 | 0 | 255 | 0 | 0 |  | 100.00±0.00 | 95.56±2.22 | 6.90±0.36 |
| 100 | 0.5 | 0.5 | 60 | 0 | 0 | 255 | 0 | 0 |  | 0.00±0.00 | 0.00±0.00 | 0.00±0.00 |
| 101 | 0.5 | 2.5 | 60 | 0 | 0 | 255 | 0 | 0 |  | 0.00±0.00 | 0.00±0.00 | 0.00±0.00 |
| 102 | 0.5 | 1.5 | 60 | 0 | 0 | 255 | 0 | 0 |  | 6.67±0.00 | 0.00±0.00 | 0.00±0.00 |
| 103 | 2.5 | 0.5 | 60 | 0 | 0 | 255 | 0 | 0 |  | 28.89±2.22 | 0.00±0.00 | 0.00±0.00 |
| 104 | 2.5 | 2.5 | 60 | 0 | 0 | 255 | 0 | 0 |  | 100.00±0.00 | 82.22±4.44 | 6.00±0.32 |
| 105 | 2.5 | 1.5 | 60 | 0 | 0 | 255 | 0 | 0 |  | 91.11±2.22 | 62.22±4.44 | 4.93±0.20 |
| 106 | 1.5 | 0.5 | 60 | 0 | 0 | 255 | 0 | 0 |  | 73.33±3.85 | 0.00±0.00 | 0.00±0.00 |
| 107 | 1.5 | 2.5 | 60 | 0 | 0 | 255 | 0 | 0 |  | 100.00±0.00 | 88.89±4.44 | 5.37±0.18 |
| **108** | **1.5** | **1.5** | **60** | **0** | **0** | **255** | **0** | **0** |  | **100.00±0.00** | **100.00±0.00** | **12.67±0.27** |
| 109 | 0.5 | 0.5 | 0 | 60 | 0 | 255 | 0 | 0 |  | 0.00±0.00 | 0.00±0.00 | 0.00±0.00 |
| 110 | 0.5 | 2.5 | 0 | 60 | 0 | 255 | 0 | 0 |  | 0.00±0.00 | 0.00±0.00 | 0.00±0.00 |
| 111 | 0.5 | 1.5 | 0 | 60 | 0 | 255 | 0 | 0 |  | 6.67±3.85 | 0.00±0.00 | 0.00±0.00 |
| 112 | 2.5 | 0.5 | 0 | 60 | 0 | 255 | 0 | 0 |  | 15.56±2.22 | 0.00±0.00 | 0.00±0.00 |
| 113 | 2.5 | 2.5 | 0 | 60 | 0 | 255 | 0 | 0 |  | 71.11±4.44 | 68.89±8.89 | 3.87±0.15 |
| 114 | 2.5 | 1.5 | 0 | 60 | 0 | 255 | 0 | 0 |  | 68.89±2.22 | 48.89±4.44 | 3.57±0.18 |
| 115 | 1.5 | 0.5 | 0 | 60 | 0 | 255 | 0 | 0 |  | 64.44±4.44 | 0.00±0.00 | 0.00±0.00 |
| 116 | 1.5 | 2.5 | 0 | 60 | 0 | 255 | 0 | 0 |  | 73.33±6.67 | 82.22±4.44 | 4.10±0.12 |
| 117 | 1.5 | 1.5 | 0 | 60 | 0 | 255 | 0 | 0 |  | 71.11±4.44 | 66.67±6.67 | 6.77±0.24 |
| 118 | 0.5 | 0.5 | 0 | 30 | 0 | 255 | 0 | 0 |  | 0.00±0.00 | 0.00±0.00 | 0.00±0.00 |
| 119 | 0.5 | 2.5 | 0 | 30 | 0 | 255 | 0 | 0 |  | 0.00±0.00 | 0.00±0.00 | 0.00±0.00 |
| 120 | 0.5 | 1.5 | 0 | 30 | 0 | 255 | 0 | 0 |  | 4.44±2.22 | 0.00±0.00 | 0.00±0.00 |
| 121 | 2.5 | 0.5 | 0 | 30 | 0 | 255 | 0 | 0 |  | 22.22±4.44 | 0.00±0.00 | 0.00±0.00 |
| 122 | 2.5 | 2.5 | 0 | 30 | 0 | 255 | 0 | 0 |  | 71.11±4.44 | 64.44±4.44 | 5.27±0.20 |
| 123 | 2.5 | 1.5 | 0 | 30 | 0 | 255 | 0 | 0 |  | 84.44±2.22 | 68.89±5.88 | 4.23±0.23 |
| 124 | 1.5 | 0.5 | 0 | 30 | 0 | 255 | 0 | 0 |  | 71.11±2.22 | 0.00±0.00 | 0.00±0.00 |
| 125 | 1.5 | 2.5 | 0 | 30 | 0 | 255 | 0 | 0 |  | 95.56±4.44 | 77.78±2.22 | 4.67±0.30 |
| 126 | 1.5 | 1.5 | 0 | 30 | 0 | 255 | 0 | 0 |  | 97.78±2.22 | 95.56±2.22 | 10.40±0.15 |
| 127 | 0.5 | 0.5 | 0 | 0 | 30 | 255 | 0 | 0 |  | 0.00±0.00 | 0.00±0.00 | 0.00±0.00 |
| 128 | 0.5 | 2.5 | 0 | 0 | 30 | 255 | 0 | 0 |  | 0.00±0.00 | 0.00±0.00 | 0.00±0.00 |
| 129 | 0.5 | 1.5 | 0 | 0 | 30 | 255 | 0 | 0 |  | 0.00±0.00 | 0.00±0.00 | 0.00±0.00 |
| 130 | 2.5 | 0.5 | 0 | 0 | 30 | 255 | 0 | 0 |  | 8.89±2.22 | 0.00±0.00 | 0.00±0.00 |
| 131 | 2.5 | 2.5 | 0 | 0 | 30 | 255 | 0 | 0 |  | 55.56±4.44 | 62.22±2.22 | 3.40±0.15 |
| 132 | 2.5 | 1.5 | 0 | 0 | 30 | 255 | 0 | 0 |  | 48.89±5.88 | 42.22±2.22 | 3.20±0.21 |
| 133 | 1.5 | 0.5 | 0 | 0 | 30 | 255 | 0 | 0 |  | 48.89±2.22 | 0.00±0.00 | 0.00±0.00 |
| 134 | 1.5 | 2.5 | 0 | 0 | 30 | 255 | 0 | 0 |  | 62.22±2.22 | 57.78±4.44 | 3.23±0.18 |
| 135 | 1.5 | 1.5 | 0 | 0 | 30 | 255 | 0 | 0 |  | 64.44±4.44 | 55.56±5.88 | 6.33±0.32 |
| 136 | 0.5 | 0.5 | 0 | 0 | 60 | 255 | 0 | 0 |  | 0.00±0.00 | 0.00±0.00 | 0.00±0.00 |
| 137 | 0.5 | 2.5 | 0 | 0 | 60 | 255 | 0 | 0 |  | 0.00±0.00 | 0.00±0.00 | 0.00±0.00 |
| 138 | 0.5 | 1.5 | 0 | 0 | 60 | 255 | 0 | 0 |  | 0.00±0.00 | 0.00±0.00 | 0.00±0.00 |
| 139 | 2.5 | 0.5 | 0 | 0 | 60 | 255 | 0 | 0 |  | 22.22±2.22 | 0.00±0.00 | 0.00±0.00 |
| 140 | 2.5 | 2.5 | 0 | 0 | 60 | 255 | 0 | 0 |  | 71.11±2.22 | 62.22±2.22 | 4.40±0.15 |
| 141 | 2.5 | 1.5 | 0 | 0 | 60 | 255 | 0 | 0 |  | 75.56±2.22 | 51.11±8.89 | 3.80±0.15 |
| 142 | 1.5 | 0.5 | 0 | 0 | 60 | 255 | 0 | 0 |  | 68.89±2.22 | 0.00±0.00 | 0.00±0.00 |
| 143 | 1.5 | 2.5 | 0 | 0 | 60 | 255 | 0 | 0 |  | 82.22±8.01 | 64.44±4.44 | 4.33±0.15 |
| 144 | 1.5 | 1.5 | 0 | 0 | 60 | 255 | 0 | 0 |  | 84.44±2.22 | 84.44±2.22 | 9.83±0.39 |
| 145 | 0.5 | 0.5 | 90 | 0 | 0 | 255 | 0 | 0 |  | 0.00±0.00 | 0.00±0.00 | 0.00±0.00 |
| 146 | 0.5 | 2.5 | 90 | 0 | 0 | 255 | 0 | 0 |  | 8.89±2.22 | 0.00±0.00 | 0.00±0.00 |
| 147 | 0.5 | 1.5 | 90 | 0 | 0 | 255 | 0 | 0 |  | 11.11±2.22 | 0.00±0.00 | 0.00±0.00 |
| 148 | 2.5 | 0.5 | 90 | 0 | 0 | 255 | 0 | 0 |  | 28.89±9.69 | 0.00±0.00 | 0.00±0.00 |
| 149 | 2.5 | 2.5 | 90 | 0 | 0 | 255 | 0 | 0 |  | 100.00±0.00 | 77.78±4.44 | 5.67±0.46 |
| 150 | 2.5 | 1.5 | 90 | 0 | 0 | 255 | 0 | 0 |  | 84.44±4.44 | 57.78±4.44 | 4.30±0.47 |
| 151 | 1.5 | 0.5 | 90 | 0 | 0 | 255 | 0 | 0 |  | 75.56±5.88 | 0.00±0.00 | 0.00±0.00 |
| 152 | 1.5 | 2.5 | 90 | 0 | 0 | 255 | 0 | 0 |  | 100.00±0.00 | 88.89±5.88 | 4.37±0.24 |
| 153 | 1.5 | 1.5 | 90 | 0 | 0 | 255 | 0 | 0 |  | 100.00±0.00 | 97.78±2.22 | 7.30±0.26 |
| 154 | 0.5 | 0.5 | 0 | 20 | 0 | 255 | 0 | 0 |  | 0.00±0.00 | 0.00±0.00 | 0.00±0.00 |
| 155 | 0.5 | 2.5 | 0 | 20 | 0 | 255 | 0 | 0 |  | 0.00±0.00 | 0.00±0.00 | 0.00±0.00 |
| 156 | 0.5 | 1.5 | 0 | 20 | 0 | 255 | 0 | 0 |  | 2.22±2.22 | 0.00±0.00 | 0.00±0.00 |
| 157 | 2.5 | 0.5 | 0 | 20 | 0 | 255 | 0 | 0 |  | 17.78±2.22 | 0.00±0.00 | 0.00±0.00 |
| 158 | 2.5 | 2.5 | 0 | 20 | 0 | 255 | 0 | 0 |  | 64.44±5.88 | 68.89±5.88 | 5.13±0.32 |
| 159 | 2.5 | 1.5 | 0 | 20 | 0 | 255 | 0 | 0 |  | 73.33±6.67 | 51.11±4.44 | 3.73±0.27 |
| 160 | 1.5 | 0.5 | 0 | 20 | 0 | 255 | 0 | 0 |  | 68.89±2.22 | 0.00±0.00 | 0.00±0.00 |
| 161 | 1.5 | 2.5 | 0 | 20 | 0 | 255 | 0 | 0 |  | 73.33±7.70 | 75.56±2.22 | 4.53±0.12 |
| 162 | 1.5 | 1.5 | 0 | 20 | 0 | 255 | 0 | 0 |  | 91.11±2.22 | 82.22±5.88 | 9.67±0.23 |
| 163 | 0.5 | 0.5 | 20 | 20 | 20 | 255 | 0 | 0 |  | 0.00±0.00 | 0.00±0.00 | 0.00±0.00 |
| 164 | 0.5 | 2.5 | 20 | 20 | 20 | 255 | 0 | 0 |  | 0.00±0.00 | 0.00±0.00 | 0.00±0.00 |
| 165 | 0.5 | 1.5 | 20 | 20 | 20 | 255 | 0 | 0 |  | 11.11±4.44 | 0.00±0.00 | 0.00±0.00 |
| 166 | 2.5 | 0.5 | 20 | 20 | 20 | 255 | 0 | 0 |  | 31.11±2.22 | 0.00±0.00 | 0.00±0.00 |
| 167 | 2.5 | 2.5 | 20 | 20 | 20 | 255 | 0 | 0 |  | 97.78±2.22 | 75.56±2.22 | 4.93±0.41 |
| 168 | 2.5 | 1.5 | 20 | 20 | 20 | 255 | 0 | 0 |  | 91.11±2.22 | 55.56±2.22 | 4.27±0.20 |
| 169 | 1.5 | 0.5 | 20 | 20 | 20 | 255 | 0 | 0 |  | 75.56±2.22 | 0.00±0.00 | 0.00±0.00 |
| 170 | 1.5 | 2.5 | 20 | 20 | 20 | 255 | 0 | 0 |  | 93.33±3.85 | 82.22±4.44 | 4.87±0.15 |
| 171 | 1.5 | 1.5 | 20 | 20 | 20 | 255 | 0 | 0 |  | 95.56±2.22 | 97.78±2.22 | 6.73±0.23 |
| 172 | 0.5 | 0.5 | 0 | 0 | 90 | 255 | 0 | 0 |  | 0.00±0.00 | 0.00±0.00 | 0.00±0.00 |
| 173 | 0.5 | 2.5 | 0 | 0 | 90 | 255 | 0 | 0 |  | 0.00±0.00 | 0.00±0.00 | 0.00±0.00 |
| 174 | 0.5 | 1.5 | 0 | 0 | 90 | 255 | 0 | 0 |  | 0.00±0.00 | 0.00±0.00 | 0.00±0.00 |
| 175 | 2.5 | 0.5 | 0 | 0 | 90 | 255 | 0 | 0 |  | 2.22±2.22 | 0.00±0.00 | 0.00±0.00 |
| 176 | 2.5 | 2.5 | 0 | 0 | 90 | 255 | 0 | 0 |  | 44.44±4.44 | 46.67±6.67 | 4.67±0.35 |
| 177 | 2.5 | 1.5 | 0 | 0 | 90 | 255 | 0 | 0 |  | 57.78±11.76 | 48.89±2.22 | 3.60±0.15 |
| 178 | 1.5 | 0.5 | 0 | 0 | 90 | 255 | 0 | 0 |  | 48.89±4.44 | 0.00±0.00 | 0.00±0.00 |
| 179 | 1.5 | 2.5 | 0 | 0 | 90 | 255 | 0 | 0 |  | 64.44±2.22 | 66.67±3.85 | 4.13±0.27 |
| 180 | 1.5 | 1.5 | 0 | 0 | 90 | 255 | 0 | 0 |  | 71.11±2.22 | 57.78±4.44 | 6.27±0.49 |
| 181 | 0.5 | 0.5 | 30 | 0 | 0 | 0 | 0 | 255 |  | 0.00±0.00 | 0.00±0.00 | 0.00±0.00 |
| 182 | 0.5 | 2.5 | 30 | 0 | 0 | 0 | 0 | 255 |  | 0.00±0.00 | 0.00±0.00 | 0.00±0.00 |
| 183 | 0.5 | 1.5 | 30 | 0 | 0 | 0 | 0 | 255 |  | 0.00±0.00 | 0.00±0.00 | 0.00±0.00 |
| 184 | 2.5 | 0.5 | 30 | 0 | 0 | 0 | 0 | 255 |  | 8.89±2.22 | 0.00±0.00 | 0.00±0.00 |
| 185 | 2.5 | 2.5 | 30 | 0 | 0 | 0 | 0 | 255 |  | 51.11±4.44 | 40.00±6.67 | 2.80±0.15 |
| 186 | 2.5 | 1.5 | 30 | 0 | 0 | 0 | 0 | 255 |  | 55.56±4.44 | 42.22±4.44 | 2.73±0.19 |
| 187 | 1.5 | 0.5 | 30 | 0 | 0 | 0 | 0 | 255 |  | 55.56±4.44 | 0.00±0.00 | 0.00±0.00 |
| 188 | 1.5 | 2.5 | 30 | 0 | 0 | 0 | 0 | 255 |  | 68.89±5.88 | 66.67±3.85 | 3.17±0.38 |
| 189 | 1.5 | 1.5 | 30 | 0 | 0 | 0 | 0 | 255 |  | 82.22±4.44 | 73.33±6.67 | 5.07±0.19 |
| 190 | 0.5 | 0.5 | 60 | 0 | 0 | 0 | 0 | 255 |  | 0.00±0.00 | 0.00±0.00 | 0.00±0.00 |
| 191 | 0.5 | 2.5 | 60 | 0 | 0 | 0 | 0 | 255 |  | 0.00±0.00 | 0.00±0.00 | 0.00±0.00 |
| 192 | 0.5 | 1.5 | 60 | 0 | 0 | 0 | 0 | 255 |  | 0.00±0.00 | 0.00±0.00 | 0.00±0.00 |
| 193 | 2.5 | 0.5 | 60 | 0 | 0 | 0 | 0 | 255 |  | 4.44±2.22 | 0.00±0.00 | 0.00±0.00 |
| 194 | 2.5 | 2.5 | 60 | 0 | 0 | 0 | 0 | 255 |  | 84.44±2.22 | 68.89±2.22 | 4.13±0.27 |
| 195 | 2.5 | 1.5 | 60 | 0 | 0 | 0 | 0 | 255 |  | 73.33±0.00 | 42.22±4.44 | 3.03±0.30 |
| 196 | 1.5 | 0.5 | 60 | 0 | 0 | 0 | 0 | 255 |  | 55.56±2.22 | 0.00±0.00 | 0.00±0.00 |
| 197 | 1.5 | 2.5 | 60 | 0 | 0 | 0 | 0 | 255 |  | 82.22±4.44 | 64.44±2.22 | 3.40±0.17 |
| 198 | 1.5 | 1.5 | 60 | 0 | 0 | 0 | 0 | 255 |  | 88.89±2.22 | 82.22±2.22 | 7.93±0.20 |
| 199 | 0.5 | 0.5 | 0 | 60 | 0 | 0 | 0 | 255 |  | 0.00±0.00 | 0.00±0.00 | 0.00±0.00 |
| 200 | 0.5 | 2.5 | 0 | 60 | 0 | 0 | 0 | 255 |  | 0.00±0.00 | 0.00±0.00 | 0.00±0.00 |
| 201 | 0.5 | 1.5 | 0 | 60 | 0 | 0 | 0 | 255 |  | 0.00±0.00 | 0.00±0.00 | 0.00±0.00 |
| 202 | 2.5 | 0.5 | 0 | 60 | 0 | 0 | 0 | 255 |  | 0.00±0.00 | 0.00±0.00 | 0.00±0.00 |
| 203 | 2.5 | 2.5 | 0 | 60 | 0 | 0 | 0 | 255 |  | 44.44±2.22 | 37.78±4.44 | 2.50±0.21 |
| 204 | 2.5 | 1.5 | 0 | 60 | 0 | 0 | 0 | 255 |  | 46.67±6.67 | 35.56±2.22 | 2.10±0.12 |
| 205 | 1.5 | 0.5 | 0 | 60 | 0 | 0 | 0 | 255 |  | 28.89±2.22 | 0.00±0.00 | 0.00±0.00 |
| 206 | 1.5 | 2.5 | 0 | 60 | 0 | 0 | 0 | 255 |  | 51.11±4.44 | 37.78±4.44 | 2.20±0.32 |
| 207 | 1.5 | 1.5 | 0 | 60 | 0 | 0 | 0 | 255 |  | 55.56±5.88 | 46.67±6.67 | 3.93±0.09 |
| 208 | 0.5 | 0.5 | 0 | 30 | 0 | 0 | 0 | 255 |  | 0.00±0.00 | 0.00±0.00 | 0.00±0.00 |
| 209 | 0.5 | 2.5 | 0 | 30 | 0 | 0 | 0 | 255 |  | 0.00±0.00 | 0.00±0.00 | 0.00±0.00 |
| 210 | 0.5 | 1.5 | 0 | 30 | 0 | 0 | 0 | 255 |  | 0.00±0.00 | 0.00±0.00 | 0.00±0.00 |
| 211 | 2.5 | 0.5 | 0 | 30 | 0 | 0 | 0 | 255 |  | 11.11±4.44 | 0.00±0.00 | 0.00±0.00 |
| 212 | 2.5 | 2.5 | 0 | 30 | 0 | 0 | 0 | 255 |  | 53.33±6.67 | 40.00±6.67 | 2.63±0.24 |
| 213 | 2.5 | 1.5 | 0 | 30 | 0 | 0 | 0 | 255 |  | 62.22±4.44 | 44.44±4.44 | 2.13±0.26 |
| 214 | 1.5 | 0.5 | 0 | 30 | 0 | 0 | 0 | 255 |  | 53.33±6.67 | 0.00±0.00 | 0.00±0.00 |
| 215 | 1.5 | 2.5 | 0 | 30 | 0 | 0 | 0 | 255 |  | 71.11±4.44 | 64.44±4.44 | 2.93±0.24 |
| 216 | 1.5 | 1.5 | 0 | 30 | 0 | 0 | 0 | 255 |  | 75.56±2.22 | 62.22±2.22 | 6.83±0.43 |
| 217 | 0.5 | 0.5 | 0 | 0 | 30 | 0 | 0 | 255 |  | 0.00±0.00 | 0.00±0.00 | 0.00±0.00 |
| 218 | 0.5 | 2.5 | 0 | 0 | 30 | 0 | 0 | 255 |  | 0.00±0.00 | 0.00±0.00 | 0.00±0.00 |
| 219 | 0.5 | 1.5 | 0 | 0 | 30 | 0 | 0 | 255 |  | 0.00±0.00 | 0.00±0.00 | 0.00±0.00 |
| 220 | 2.5 | 0.5 | 0 | 0 | 30 | 0 | 0 | 255 |  | 0.00±0.00 | 0.00±0.00 | 0.00±0.00 |
| 221 | 2.5 | 2.5 | 0 | 0 | 30 | 0 | 0 | 255 |  | 28.89±2.22 | 22.22±2.22 | 2.27±0.20 |
| 222 | 2.5 | 1.5 | 0 | 0 | 30 | 0 | 0 | 255 |  | 37.78±2.22 | 31.11±2.22 | 1.70±0.10 |
| 223 | 1.5 | 0.5 | 0 | 0 | 30 | 0 | 0 | 255 |  | 17.78±2.22 | 0.00±0.00 | 0.00±0.00 |
| 224 | 1.5 | 2.5 | 0 | 0 | 30 | 0 | 0 | 255 |  | 53.33±6.67 | 46.67±6.67 | 2.43±0.07 |
| 225 | 1.5 | 1.5 | 0 | 0 | 30 | 0 | 0 | 255 |  | 48.89±2.22 | 35.56±2.22 | 4.43±0.33 |
| 226 | 0.5 | 0.5 | 0 | 0 | 60 | 0 | 0 | 255 |  | 0.00±0.00 | 0.00±0.00 | 0.00±0.00 |
| 227 | 0.5 | 2.5 | 0 | 0 | 60 | 0 | 0 | 255 |  | 0.00±0.00 | 0.00±0.00 | 0.00±0.00 |
| 228 | 0.5 | 1.5 | 0 | 0 | 60 | 0 | 0 | 255 |  | 0.00±0.00 | 0.00±0.00 | 0.00±0.00 |
| 229 | 2.5 | 0.5 | 0 | 0 | 60 | 0 | 0 | 255 |  | 2.22±2.22 | 0.00±0.00 | 0.00±0.00 |
| 230 | 2.5 | 2.5 | 0 | 0 | 60 | 0 | 0 | 255 |  | 48.89±2.22 | 40.00±3.85 | 2.67±0.23 |
| 231 | 2.5 | 1.5 | 0 | 0 | 60 | 0 | 0 | 255 |  | 51.11±2.22 | 37.78±4.44 | 1.33±0.15 |
| 232 | 1.5 | 0.5 | 0 | 0 | 60 | 0 | 0 | 255 |  | 31.11±4.44 | 0.00±0.00 | 0.00±0.00 |
| 233 | 1.5 | 2.5 | 0 | 0 | 60 | 0 | 0 | 255 |  | 64.44±5.88 | 57.78±4.44 | 2.93±0.09 |
| 234 | 1.5 | 1.5 | 0 | 0 | 60 | 0 | 0 | 255 |  | 68.89±2.22 | 62.22±2.22 | 6.90±0.50 |
| 235 | 0.5 | 0.5 | 90 | 0 | 0 | 0 | 0 | 255 |  | 0.00±0.00 | 0.00±0.00 | 0.00±0.00 |
| 236 | 0.5 | 2.5 | 90 | 0 | 0 | 0 | 0 | 255 |  | 0.00±0.00 | 0.00±0.00 | 0.00±0.00 |
| 237 | 0.5 | 1.5 | 90 | 0 | 0 | 0 | 0 | 255 |  | 0.00±0.00 | 0.00±0.00 | 0.00±0.00 |
| 238 | 2.5 | 0.5 | 90 | 0 | 0 | 0 | 0 | 255 |  | 2.22±2.22 | 0.00±0.00 | 0.00±0.00 |
| 239 | 2.5 | 2.5 | 90 | 0 | 0 | 0 | 0 | 255 |  | 75.56±2.22 | 57.78±4.44 | 3.53±0.23 |
| 240 | 2.5 | 1.5 | 90 | 0 | 0 | 0 | 0 | 255 |  | 68.89±2.22 | 44.44±4.44 | 2.60±0.29 |
| 241 | 1.5 | 0.5 | 90 | 0 | 0 | 0 | 0 | 255 |  | 51.11±4.44 | 0.00±0.00 | 0.00±0.00 |
| 242 | 1.5 | 2.5 | 90 | 0 | 0 | 0 | 0 | 255 |  | 73.33±6.67 | 57.78±5.88 | 3.20±0.21 |
| 243 | 1.5 | 1.5 | 90 | 0 | 0 | 0 | 0 | 255 |  | 77.78±4.44 | 71.11±4.44 | 4.63±0.24 |
| 244 | 0.5 | 0.5 | 0 | 20 | 0 | 0 | 0 | 255 |  | 0.00±0.00 | 0.00±0.00 | 0.00±0.00 |
| 245 | 0.5 | 2.5 | 0 | 20 | 0 | 0 | 0 | 255 |  | 0.00±0.00 | 0.00±0.00 | 0.00±0.00 |
| 246 | 0.5 | 1.5 | 0 | 20 | 0 | 0 | 0 | 255 |  | 0.00±0.00 | 0.00±0.00 | 0.00±0.00 |
| 247 | 2.5 | 0.5 | 0 | 20 | 0 | 0 | 0 | 255 |  | 4.44±4.44 | 0.00±0.00 | 0.00±0.00 |
| 248 | 2.5 | 2.5 | 0 | 20 | 0 | 0 | 0 | 255 |  | 42.22±2.22 | 42.22±8.89 | 3.93±0.38 |
| 249 | 2.5 | 1.5 | 0 | 20 | 0 | 0 | 0 | 255 |  | 53.33±6.67 | 46.67±6.67 | 2.73±0.19 |
| 250 | 1.5 | 0.5 | 0 | 20 | 0 | 0 | 0 | 255 |  | 57.78±2.22 | 0.00±0.00 | 0.00±0.00 |
| 251 | 1.5 | 2.5 | 0 | 20 | 0 | 0 | 0 | 255 |  | 62.22±4.44 | 57.78±2.22 | 2.60±0.29 |
| 252 | 1.5 | 1.5 | 0 | 20 | 0 | 0 | 0 | 255 |  | 68.89±2.22 | 64.44±2.22 | 7.63±0.24 |
| 253 | 0.5 | 0.5 | 20 | 20 | 20 | 0 | 0 | 255 |  | 0.00±0.00 | 0.00±0.00 | 0.00±0.00 |
| 254 | 0.5 | 2.5 | 20 | 20 | 20 | 0 | 0 | 255 |  | 0.00±0.00 | 0.00±0.00 | 0.00±0.00 |
| 255 | 0.5 | 1.5 | 20 | 20 | 20 | 0 | 0 | 255 |  | 0.00±0.00 | 0.00±0.00 | 0.00±0.00 |
| 256 | 2.5 | 0.5 | 20 | 20 | 20 | 0 | 0 | 255 |  | 6.67±0.00 | 0.00±0.00 | 0.00±0.00 |
| 257 | 2.5 | 2.5 | 20 | 20 | 20 | 0 | 0 | 255 |  | 55.56±2.22 | 44.44±2.22 | 1.97±0.29 |
| 258 | 2.5 | 1.5 | 20 | 20 | 20 | 0 | 0 | 255 |  | 62.22±2.22 | 42.22±2.22 | 1.73±0.45 |
| 259 | 1.5 | 0.5 | 20 | 20 | 20 | 0 | 0 | 255 |  | 55.56±2.22 | 0.00±0.00 | 0.00±0.00 |
| 260 | 1.5 | 2.5 | 20 | 20 | 20 | 0 | 0 | 255 |  | 82.22±2.22 | 71.11±4.44 | 2.03±0.29 |
| 261 | 1.5 | 1.5 | 20 | 20 | 20 | 0 | 0 | 255 |  | 84.44±4.44 | 73.33±6.67 | 4.00±0.38 |
| 262 | 0.5 | 0.5 | 0 | 0 | 90 | 0 | 0 | 255 |  | 0.00±0.00 | 0.00±0.00 | 0.00±0.00 |
| 263 | 0.5 | 2.5 | 0 | 0 | 90 | 0 | 0 | 255 |  | 0.00±0.00 | 0.00±0.00 | 0.00±0.00 |
| 264 | 0.5 | 1.5 | 0 | 0 | 90 | 0 | 0 | 255 |  | 0.00±0.00 | 0.00±0.00 | 0.00±0.00 |
| 265 | 2.5 | 0.5 | 0 | 0 | 90 | 0 | 0 | 255 |  | 0.00±0.00 | 0.00±0.00 | 0.00±0.00 |
| 266 | 2.5 | 2.5 | 0 | 0 | 90 | 0 | 0 | 255 |  | 22.22±2.22 | 15.56±2.22 | 2.20±0.21 |
| 267 | 2.5 | 1.5 | 0 | 0 | 90 | 0 | 0 | 255 |  | 48.89±4.44 | 24.44±4.44 | 1.40±0.15 |
| 268 | 1.5 | 0.5 | 0 | 0 | 90 | 0 | 0 | 255 |  | 31.11±4.44 | 0.00±0.00 | 0.00±0.00 |
| 269 | 1.5 | 2.5 | 0 | 0 | 90 | 0 | 0 | 255 |  | 42.22±4.44 | 31.11±5.88 | 1.60±0.29 |
| 270 | 1.5 | 1.5 | 0 | 0 | 90 | 0 | 0 | 255 |  | 40.00±6.67 | 35.56±2.22 | 2.87±0.20 |
| 271 | 0.5 | 0.5 | 30 | 0 | 0 | 255 | 255 | 255 |  | 0.00±0.00 | 0.00±0.00 | 0.00±0.00 |
| 272 | 0.5 | 2.5 | 30 | 0 | 0 | 255 | 255 | 255 |  | 0.00±0.00 | 0.00±0.00 | 0.00±0.00 |
| 273 | 0.5 | 1.5 | 30 | 0 | 0 | 255 | 255 | 255 |  | 4.44±2.22 | 0.00±0.00 | 0.00±0.00 |
| 274 | 2.5 | 0.5 | 30 | 0 | 0 | 255 | 255 | 255 |  | 17.78±4.44 | 0.00±0.00 | 0.00±0.00 |
| 275 | 2.5 | 2.5 | 30 | 0 | 0 | 255 | 255 | 255 |  | 77.78±4.44 | 57.78±4.44 | 4.27±0.66 |
| 276 | 2.5 | 1.5 | 30 | 0 | 0 | 255 | 255 | 255 |  | 62.22±9.69 | 44.44±5.88 | 3.33±0.45 |
| 277 | 1.5 | 0.5 | 30 | 0 | 0 | 255 | 255 | 255 |  | 62.22±2.22 | 0.00±0.00 | 0.00±0.00 |
| 278 | 1.5 | 2.5 | 30 | 0 | 0 | 255 | 255 | 255 |  | 75.56±8.89 | 73.33±6.67 | 3.60±0.29 |
| 279 | 1.5 | 1.5 | 30 | 0 | 0 | 255 | 255 | 255 |  | 91.11±4.44 | 88.89±2.22 | 6.13±0.20 |
| 280 | 0.5 | 0.5 | 60 | 0 | 0 | 255 | 255 | 255 |  | 0.00±0.00 | 0.00±0.00 | 0.00±0.00 |
| 281 | 0.5 | 2.5 | 60 | 0 | 0 | 255 | 255 | 255 |  | 0.00±0.00 | 0.00±0.00 | 0.00±0.00 |
| 282 | 0.5 | 1.5 | 60 | 0 | 0 | 255 | 255 | 255 |  | 2.22±2.22 | 0.00±0.00 | 0.00±0.00 |
| 283 | 2.5 | 0.5 | 60 | 0 | 0 | 255 | 255 | 255 |  | 22.22±4.44 | 0.00±0.00 | 0.00±0.00 |
| 284 | 2.5 | 2.5 | 60 | 0 | 0 | 255 | 255 | 255 |  | 100.00±0.00 | 71.11±4.44 | 5.13±0.58 |
| 285 | 2.5 | 1.5 | 60 | 0 | 0 | 255 | 255 | 255 |  | 77.78±4.44 | 44.44±5.88 | 3.83±0.28 |
| 286 | 1.5 | 0.5 | 60 | 0 | 0 | 255 | 255 | 255 |  | 71.11±2.22 | 0.00±0.00 | 0.00±0.00 |
| 287 | 1.5 | 2.5 | 60 | 0 | 0 | 255 | 255 | 255 |  | 95.56±2.22 | 77.78±5.88 | 4.17±0.18 |
| 288 | 1.5 | 1.5 | 60 | 0 | 0 | 255 | 255 | 255 |  | 97.78±2.22 | 97.78±2.22 | 9.53±0.46 |
| 289 | 0.5 | 0.5 | 0 | 60 | 0 | 255 | 255 | 255 |  | 0.00±0.00 | 0.00±0.00 | 0.00±0.00 |
| 290 | 0.5 | 2.5 | 0 | 60 | 0 | 255 | 255 | 255 |  | 0.00±0.00 | 0.00±0.00 | 0.00±0.00 |
| 291 | 0.5 | 1.5 | 0 | 60 | 0 | 255 | 255 | 255 |  | 0.00±0.00 | 0.00±0.00 | 0.00±0.00 |
| 292 | 2.5 | 0.5 | 0 | 60 | 0 | 255 | 255 | 255 |  | 4.44±2.22 | 0.00±0.00 | 0.00±0.00 |
| 293 | 2.5 | 2.5 | 0 | 60 | 0 | 255 | 255 | 255 |  | 55.56±4.44 | 51.11±4.44 | 2.90±0.26 |
| 294 | 2.5 | 1.5 | 0 | 60 | 0 | 255 | 255 | 255 |  | 53.33±7.70 | 37.78±4.44 | 2.30±0.21 |
| 295 | 1.5 | 0.5 | 0 | 60 | 0 | 255 | 255 | 255 |  | 42.22±4.44 | 0.00±0.00 | 0.00±0.00 |
| 296 | 1.5 | 2.5 | 0 | 60 | 0 | 255 | 255 | 255 |  | 64.44±4.44 | 55.56±11.11 | 2.80±0.10 |
| 297 | 1.5 | 1.5 | 0 | 60 | 0 | 255 | 255 | 255 |  | 64.44±4.44 | 62.22±8.89 | 4.87±0.34 |
| 298 | 0.5 | 0.5 | 0 | 30 | 0 | 255 | 255 | 255 |  | 0.00±0.00 | 0.00±0.00 | 0.00±0.00 |
| 299 | 0.5 | 2.5 | 0 | 30 | 0 | 255 | 255 | 255 |  | 0.00±0.00 | 0.00±0.00 | 0.00±0.00 |
| 300 | 0.5 | 1.5 | 0 | 30 | 0 | 255 | 255 | 255 |  | 0.00±0.00 | 0.00±0.00 | 0.00±0.00 |
| 301 | 2.5 | 0.5 | 0 | 30 | 0 | 255 | 255 | 255 |  | 15.56±4.44 | 0.00±0.00 | 0.00±0.00 |
| 302 | 2.5 | 2.5 | 0 | 30 | 0 | 255 | 255 | 255 |  | 66.67±6.67 | 55.56±4.44 | 3.57±0.20 |
| 303 | 2.5 | 1.5 | 0 | 30 | 0 | 255 | 255 | 255 |  | 75.56±4.44 | 42.22±4.44 | 2.50±0.21 |
| 304 | 1.5 | 0.5 | 0 | 30 | 0 | 255 | 255 | 255 |  | 64.44±2.22 | 0.00±0.00 | 0.00±0.00 |
| 305 | 1.5 | 2.5 | 0 | 30 | 0 | 255 | 255 | 255 |  | 88.89±2.22 | 68.89±4.44 | 3.87±0.38 |
| 306 | 1.5 | 1.5 | 0 | 30 | 0 | 255 | 255 | 255 |  | 88.89±2.22 | 80.00±6.67 | 9.23±0.32 |
| 307 | 0.5 | 0.5 | 0 | 0 | 30 | 255 | 255 | 255 |  | 0.00±0.00 | 0.00±0.00 | 0.00±0.00 |
| 308 | 0.5 | 2.5 | 0 | 0 | 30 | 255 | 255 | 255 |  | 0.00±0.00 | 0.00±0.00 | 0.00±0.00 |
| 309 | 0.5 | 1.5 | 0 | 0 | 30 | 255 | 255 | 255 |  | 0.00±0.00 | 0.00±0.00 | 0.00±0.00 |
| 310 | 2.5 | 0.5 | 0 | 0 | 30 | 255 | 255 | 255 |  | 2.22±2.22 | 0.00±0.00 | 0.00±0.00 |
| 311 | 2.5 | 2.5 | 0 | 0 | 30 | 255 | 255 | 255 |  | 42.22±2.22 | 48.89±2.22 | 2.87±0.32 |
| 312 | 2.5 | 1.5 | 0 | 0 | 30 | 255 | 255 | 255 |  | 42.22±4.44 | 35.56±2.22 | 1.93±0.20 |
| 313 | 1.5 | 0.5 | 0 | 0 | 30 | 255 | 255 | 255 |  | 37.78±2.22 | 0.00±0.00 | 0.00±0.00 |
| 314 | 1.5 | 2.5 | 0 | 0 | 30 | 255 | 255 | 255 |  | 55.56±8.89 | 44.44±11.11 | 2.50±0.21 |
| 315 | 1.5 | 1.5 | 0 | 0 | 30 | 255 | 255 | 255 |  | 51.11±4.44 | 42.22±8.89 | 4.93±0.35 |
| 316 | 0.5 | 0.5 | 0 | 0 | 60 | 255 | 255 | 255 |  | 0.00±0.00 | 0.00±0.00 | 0.00±0.00 |
| 317 | 0.5 | 2.5 | 0 | 0 | 60 | 255 | 255 | 255 |  | 0.00±0.00 | 0.00±0.00 | 0.00±0.00 |
| 318 | 0.5 | 1.5 | 0 | 0 | 60 | 255 | 255 | 255 |  | 0.00±0.00 | 0.00±0.00 | 0.00±0.00 |
| 319 | 2.5 | 0.5 | 0 | 0 | 60 | 255 | 255 | 255 |  | 8.89±2.22 | 0.00±0.00 | 0.00±0.00 |
| 320 | 2.5 | 2.5 | 0 | 0 | 60 | 255 | 255 | 255 |  | 62.22±2.22 | 42.22±2.22 | 3.07±0.09 |
| 321 | 2.5 | 1.5 | 0 | 0 | 60 | 255 | 255 | 255 |  | 62.22±2.22 | 42.22±2.22 | 2.30±0.12 |
| 322 | 1.5 | 0.5 | 0 | 0 | 60 | 255 | 255 | 255 |  | 55.56±4.44 | 0.00±0.00 | 0.00±0.00 |
| 323 | 1.5 | 2.5 | 0 | 0 | 60 | 255 | 255 | 255 |  | 77.78±4.44 | 60.00±6.67 | 3.40±0.15 |
| 324 | 1.5 | 1.5 | 0 | 0 | 60 | 255 | 255 | 255 |  | 71.11±2.22 | 71.11±4.44 | 7.93±0.20 |
| 325 | 0.5 | 0.5 | 90 | 0 | 0 | 255 | 255 | 255 |  | 0.00±0.00 | 0.00±0.00 | 0.00±0.00 |
| 326 | 0.5 | 2.5 | 90 | 0 | 0 | 255 | 255 | 255 |  | 2.22±2.22 | 0.00±0.00 | 0.00±0.00 |
| 327 | 0.5 | 1.5 | 90 | 0 | 0 | 255 | 255 | 255 |  | 6.67±0.00 | 0.00±0.00 | 0.00±0.00 |
| 328 | 2.5 | 0.5 | 90 | 0 | 0 | 255 | 255 | 255 |  | 13.33±6.67 | 0.00±0.00 | 0.00±0.00 |
| 329 | 2.5 | 2.5 | 90 | 0 | 0 | 255 | 255 | 255 |  | 88.89±2.22 | 64.44±4.44 | 3.87±0.43 |
| 330 | 2.5 | 1.5 | 90 | 0 | 0 | 255 | 255 | 255 |  | 77.78±2.22 | 51.11±4.44 | 3.50±0.31 |
| 331 | 1.5 | 0.5 | 90 | 0 | 0 | 255 | 255 | 255 |  | 68.89±2.22 | 0.00±0.00 | 0.00±0.00 |
| 332 | 1.5 | 2.5 | 90 | 0 | 0 | 255 | 255 | 255 |  | 86.67±7.70 | 73.33±6.67 | 3.67±0.15 |
| 333 | 1.5 | 1.5 | 90 | 0 | 0 | 255 | 255 | 255 |  | 88.89±2.22 | 82.22±2.22 | 6.03±0.22 |
| 334 | 0.5 | 0.5 | 0 | 20 | 0 | 255 | 255 | 255 |  | 0.00±0.00 | 0.00±0.00 | 0.00±0.00 |
| 335 | 0.5 | 2.5 | 0 | 20 | 0 | 255 | 255 | 255 |  | 0.00±0.00 | 0.00±0.00 | 0.00±0.00 |
| 336 | 0.5 | 1.5 | 0 | 20 | 0 | 255 | 255 | 255 |  | 2.22±2.22 | 0.00±0.00 | 0.00±0.00 |
| 337 | 2.5 | 0.5 | 0 | 20 | 0 | 255 | 255 | 255 |  | 11.11±4.44 | 0.00±0.00 | 0.00±0.00 |
| 338 | 2.5 | 2.5 | 0 | 20 | 0 | 255 | 255 | 255 |  | 55.56±2.22 | 53.33±6.67 | 4.40±0.81 |
| 339 | 2.5 | 1.5 | 0 | 20 | 0 | 255 | 255 | 255 |  | 64.44±5.88 | 44.44±2.22 | 3.00±0.26 |
| 340 | 1.5 | 0.5 | 0 | 20 | 0 | 255 | 255 | 255 |  | 62.22±2.22 | 0.00±0.00 | 0.00±0.00 |
| 341 | 1.5 | 2.5 | 0 | 20 | 0 | 255 | 255 | 255 |  | 64.44±5.88 | 68.89±2.22 | 3.37±0.15 |
| 342 | 1.5 | 1.5 | 0 | 20 | 0 | 255 | 255 | 255 |  | 82.22±4.44 | 73.33±6.67 | 8.00±0.38 |
| 343 | 0.5 | 0.5 | 20 | 20 | 20 | 255 | 255 | 255 |  | 0.00±0.00 | 0.00±0.00 | 0.00±0.00 |
| 344 | 0.5 | 2.5 | 20 | 20 | 20 | 255 | 255 | 255 |  | 0.00±0.00 | 0.00±0.00 | 0.00±0.00 |
| 345 | 0.5 | 1.5 | 20 | 20 | 20 | 255 | 255 | 255 |  | 4.44±2.22 | 0.00±0.00 | 0.00±0.00 |
| 346 | 2.5 | 0.5 | 20 | 20 | 20 | 255 | 255 | 255 |  | 15.56±4.44 | 0.00±0.00 | 0.00±0.00 |
| 347 | 2.5 | 2.5 | 20 | 20 | 20 | 255 | 255 | 255 |  | 91.11±2.22 | 64.44±2.22 | 3.63±0.34 |
| 348 | 2.5 | 1.5 | 20 | 20 | 20 | 255 | 255 | 255 |  | 77.78±2.22 | 48.89±2.22 | 3.50±0.23 |
| 349 | 1.5 | 0.5 | 20 | 20 | 20 | 255 | 255 | 255 |  | 64.44±4.44 | 0.00±0.00 | 0.00±0.00 |
| 350 | 1.5 | 2.5 | 20 | 20 | 20 | 255 | 255 | 255 |  | 88.89±2.22 | 75.56±2.22 | 2.70±0.10 |
| 351 | 1.5 | 1.5 | 20 | 20 | 20 | 255 | 255 | 255 |  | 91.11±2.22 | 88.89±4.44 | 5.30±0.23 |
| 352 | 0.5 | 0.5 | 0 | 0 | 90 | 255 | 255 | 255 |  | 0.00±0.00 | 0.00±0.00 | 0.00±0.00 |
| 353 | 0.5 | 2.5 | 0 | 0 | 90 | 255 | 255 | 255 |  | 0.00±0.00 | 0.00±0.00 | 0.00±0.00 |
| 354 | 0.5 | 1.5 | 0 | 0 | 90 | 255 | 255 | 255 |  | 0.00±0.00 | 0.00±0.00 | 0.00±0.00 |
| 355 | 2.5 | 0.5 | 0 | 0 | 90 | 255 | 255 | 255 |  | 0.00±0.00 | 0.00±0.00 | 0.00±0.00 |
| 356 | 2.5 | 2.5 | 0 | 0 | 90 | 255 | 255 | 255 |  | 35.56±2.22 | 31.11±5.88 | 3.17±0.26 |
| 357 | 2.5 | 1.5 | 0 | 0 | 90 | 255 | 255 | 255 |  | 51.11±4.44 | 31.11±4.44 | 2.43±0.27 |
| 358 | 1.5 | 0.5 | 0 | 0 | 90 | 255 | 255 | 255 |  | 44.44±2.22 | 0.00±0.00 | 0.00±0.00 |
| 359 | 1.5 | 2.5 | 0 | 0 | 90 | 255 | 255 | 255 |  | 51.11±8.89 | 42.22±4.44 | 2.27±0.17 |
| 360 | 1.5 | 1.5 | 0 | 0 | 90 | 255 | 255 | 255 |  | 48.89±9.69 | 46.67±7.70 | 4.07±0.32 |
| Values in each column represent means ±SE. | | | | | | | | | | | | |
